# The efficacy of dihydroartemisinin-piperaquine and artemether-lumefantrine with and without primaquine on *Plasmodium vivax* recurrence: A systematic review and individual patient data meta-analysis

**DOI:** 10.1371/journal.pmed.1002928

**Published:** 2019-10-04

**Authors:** Robert J. Commons, Julie A. Simpson, Kamala Thriemer, Tesfay Abreha, Ishag Adam, Nicholas M. Anstey, Ashenafi Assefa, Ghulam R. Awab, J. Kevin Baird, Bridget E. Barber, Cindy S. Chu, Prabin Dahal, André Daher, Timothy M. E. Davis, Arjen M. Dondorp, Matthew J. Grigg, Georgina S. Humphreys, Jimee Hwang, Harin Karunajeewa, Moses Laman, Kartini Lidia, Brioni R. Moore, Ivo Mueller, Francois Nosten, Ayodhia P. Pasaribu, Dhelio B. Pereira, Aung P. Phyo, Jeanne R. Poespoprodjo, Carol H. Sibley, Kasia Stepniewska, Inge Sutanto, Guy Thwaites, Tran T. Hien, Nicholas J. White, Timothy William, Charles J. Woodrow, Philippe J. Guerin, Ric N. Price

**Affiliations:** 1 Global Health Division, Menzies School of Health Research and Charles Darwin University, Darwin, Northern Territory, Australia; 2 WorldWide Antimalarial Resistance Network (WWARN), Clinical module, Darwin, Northern Territory, Australia; 3 Centre for Epidemiology and Biostatistics, Melbourne School of Population and Global Health, The University of Melbourne, Melbourne, Victoria, Australia; 4 ICAP, Columbia University Mailman School of Public Health, Addis Ababa, Ethiopia; 5 Faculty of Medicine, University of Khartoum, Khartoum, Sudan; 6 Malaria and Neglected Tropical Diseases Research Team, Bacterial, Parasitic, Zoonotic Diseases Research Directorate, Ethiopian Public Health Institute, Addis Ababa, Ethiopia; 7 Mahidol-Oxford Tropical Medicine Research Unit (MORU), Faculty of Tropical Medicine, Mahidol University, Bangkok, Thailand; 8 Nangarhar Medical Faculty, Nangarhar University, Jalalabad, Afghanistan; 9 Eijkman-Oxford Clinical Research Unit, Jakarta, Indonesia; 10 Centre for Tropical Medicine and Global Health, Nuffield Department of Clinical Medicine, University of Oxford, Oxford, United Kingdom; 11 Infectious Diseases Society Sabah-Menzies School of Health Research Clinical Research Unit, Kota Kinabalu, Sabah, Malaysia; 12 Shoklo Malaria Research Unit, Mahidol-Oxford Tropical Medicine Research Unit, Faculty of Tropical Medicine, Mahidol University, Mae Sot, Thailand; 13 WorldWide Antimalarial Resistance Network (WWARN), Oxford, United Kingdom; 14 Institute of Drug Technology (Farmanguinhos), Oswaldo Cruz Foundation (FIOCRUZ), Rio de Janeiro, Brazil; 15 Vice‑presidency of Research and Reference Laboratories, Oswaldo Cruz Foundation (FIOCRUZ), Rio de Janeiro, Brazil; 16 Liverpool School of Tropical Medicine, Liverpool, United Kingdom; 17 Medical School, The University of Western Australia, Fremantle Hospital Unit, Fremantle, Western Australia, Australia; 18 U.S. President's Malaria Initiative, Malaria Branch, U.S. Centers for Disease Control and Prevention, Atlanta, Georgia, United States of America; 19 Global Health Group, University of California San Francisco, San Francisco, California, United States of America; 20 Division of Population Health and Immunity, Walter and Eliza Hall Institute, Parkville, Victoria, Australia; 21 Western Centre for Health Research and Education, Western Health, Melbourne, Victoria, Australia; 22 Papua New Guinea Institute of Medical Research, Madang, Madang Province, Papua New Guinea; 23 The Department of Pharmacology and Therapy, Faculty of Medicine, Nusa Cendana University, Kupang, Indonesia; 24 School of Pharmacy and Biomedical Sciences, Curtin University, Perth, Western Australia, Australia; 25 Department of Medical Biology, University of Melbourne, Melbourne, Victoria, Australia; 26 Parasites and Insect Vectors Department, Institut Pasteur, Paris, France; 27 Medical Faculty, Universitas Sumatera Utara, Medan, North Sumatera, Indonesia; 28 Centro de Pesquisa em Medicina Tropical de Rondônia (CEPEM), Porto Velho, Rondônia, Brazil; 29 Universidade Federal de Rondônia (UNIR), Porto Velho, Rondônia, Brazil; 30 Mimika District Hospital, Timika, Indonesia; 31 Timika Malaria Research Programme, Papuan Health and Community Development Foundation, Timika, Indonesia; 32 Paediatric Research Office, Department of Child Health, Faculty of Medicine, Public Health and Nursing, Universitas Gadjah Mada/Dr. Sardjito Hospital, Yogyakarta, Indonesia; 33 Department of Genome Sciences, University of Washington, Seattle, Washington, United States of America; 34 Department of Parasitology, Faculty of Medicine, University of Indonesia, Jakarta, Indonesia; 35 Oxford University Clinical Research Unit, Ho Chi Minh City, Vietnam; 36 Gleneagles Hospital, Kota Kinabalu, Sabah, Malaysia; ISGlobal, SPAIN

## Abstract

**Background:**

Artemisinin-based combination therapy (ACT) is recommended for uncomplicated *Plasmodium vivax* malaria in areas of emerging chloroquine resistance. We undertook a systematic review and individual patient data meta-analysis to compare the efficacies of dihydroartemisinin-piperaquine (DP) and artemether-lumefantrine (AL) with or without primaquine (PQ) on the risk of recurrent *P*. *vivax*.

**Methods and findings:**

Clinical efficacy studies of uncomplicated *P*. *vivax* treated with DP or AL and published between January 1, 2000, and January 31, 2018, were identified by conducting a systematic review registered with the International Prospective Register of Systematic Reviews (PROSPERO): CRD42016053310. Investigators of eligible studies were invited to contribute individual patient data that were pooled using standardised methodology. The effect of mg/kg dose of piperaquine/lumefantrine, ACT administered, and PQ on the rate of *P*. *vivax* recurrence between days 7 and 42 after starting treatment were investigated by Cox regression analyses according to an a priori analysis plan. Secondary outcomes were the risk of recurrence assessed on days 28 and 63. Nineteen studies enrolling 2,017 patients were included in the analysis. The risk of recurrent *P*. *vivax* at day 42 was significantly higher in the 384 patients treated with AL alone (44.0%, 95% confidence interval [CI] 38.7–49.8) compared with the 812 patients treated with DP alone (9.3%, 95% CI 7.1–12.2): adjusted hazard ratio (AHR) 12.63 (95% CI 6.40–24.92), *p* < 0.001. The rates of recurrence assessed at days 42 and 63 were associated inversely with the dose of piperaquine: AHRs (95% CI) for every 5-mg/kg increase 0.63 (0.48–0.84), *p* = 0.0013 and 0.83 (0.73–0.94), *p* = 0.0033, respectively. The dose of lumefantrine was not significantly associated with the rate of recurrence (1.07 for every 5-mg/kg increase, 95% CI 0.99–1.16, *p* = 0.0869). In a post hoc analysis, in patients with symptomatic recurrence after AL, the mean haemoglobin increased 0.13 g/dL (95% CI 0.01–0.26) for every 5 days that recurrence was delayed, *p* = 0.0407. Coadministration of PQ reduced substantially the rate of recurrence assessed at day 42 after AL (AHR = 0.20, 95% CI 0.10–0.41, *p* < 0.001) and at day 63 after DP (AHR = 0.08, 95% CI 0.01–0.70, *p* = 0.0233). Results were limited by follow-up of patients to 63 days or less and nonrandomised treatment groups.

**Conclusions:**

In this study, we observed the risk of *P*. *vivax* recurrence at day 42 to be significantly lower following treatment with DP compared with AL, reflecting the longer period of post-treatment prophylaxis; this risk was reduced substantially by coadministration with PQ. We found that delaying *P*. *vivax* recurrence was associated with a small but significant improvement in haemoglobin. These results highlight the benefits of PQ radical cure and also the provision of blood-stage antimalarial agents with prolonged post-treatment prophylaxis.

## Abstract 2

In this systematic review and individual patient data meta-analysis, Robert Commons and colleagues compare the clinical efficacy of different artemisinin-based combination therapies for treating Plasmodium vivax malaria.

## Author summary

**Why was this study done?:**

- The susceptibility of *Plasmodium vivax* to chloroquine is decreasing in many malaria-endemic locations.
- Artemisinin-based combination therapies (ACTs) are recommended in areas of emerging chloroquine resistance; however, the optimal ACT is not clear.
- Knowledge of the comparative advantages and disadvantages of different ACTs will help guide national policy makers.

**What did the researchers do and find?:**

- Following a systematic review, individual data from 2,017 patients were pooled from 19 studies undertaken between January 1, 2000, and January 31, 2018.
- Cox regression analysis showed that the risk of recurrence at day 42 was 12-fold greater following treatment with artemether-lumefantrine (AL) alone compared with dihydroartemisinin-piperaquine (DP), although by day 63 the risk of recurrence following DP was also high.
- For every 5-mg/kg increase in the dose of piperaquine, the risk of recurrence at day 42 fell by 37%.
- A delay in the time to symptomatic recurrence was associated with an increase in haemoglobin.
- Coadministration with primaquine reduced the risk of recurrence at day 42 after AL by 80% and at day 63 after DP by 92%.

**What do these findings mean?:**

- There is a high risk of recurrence following AL or DP unless they are combined with primaquine.
- Compared with AL, patients treated with DP have a reduced risk of early *P*. *vivax* recurrence.
- Delaying the time to recurrence was associated with greater haematological recovery, and this has potential to prevent morbidity related to multiple rapid recurrences by preventing a cumulative risk of anaemia.

## Introduction

Declining efficacy of chloroquine against *Plasmodium vivax* has been reported at varying degrees across much of the vivax-endemic world [1]. This erosion of efficacy is observed initially as breakthrough to patency of the first relapse in tropical strains within 28 days [2–4]. Each episode of recurrent patent parasitaemia is associated with a risk of morbidity, a cumulative risk of anaemia, and increased transmission, threatening malaria control and elimination efforts [5–8].

Potential options to respond to declining chloroquine efficacy include prescribing a higher dose of chloroquine or ensuring that radical cure is provided—i.e., combining chloroquine with primaquine (PQ) [9]. Alternatively, some countries have changed their national antimalarial guidelines to recommend artemisinin-based combination therapy (ACT) for both *P*. *falciparum* and *P*. *vivax* malaria [10]. The ACTs adopted differ, with Cambodia and Indonesia using dihydroartemisinin-piperaquine (DP) and Papua New Guinea, Solomon Islands, and Vanuatu using artemether-lumefantrine (AL) [11].

Relapses can be prevented by combining blood schizontocidal treatment and PQ, an 8-aminoquinoline with activity against the dormant liver stages of *P*. *vivax* [2]. In tropical areas, relapses can occur within weeks of the initial infection, leading to a further episode of haemolysis, prior to complete haematological recovery from the first episode [12,13]. Partner drugs in ACTs vary significantly in their terminal elimination half-lives, with the more rapidly eliminated lumefantrine providing a shorter period of post-treatment prophylaxis against early reinfections and relapses compared with the more slowly eliminated piperaquine [6,14,15].

To inform decisions regarding optimal treatment policy in areas of emerging chloroquine-resistant *P*. *vivax*, we conducted a systematic review and individual patient data meta-analysis to compare the efficacy of AL and DP for treatment of uncomplicated *P*. *vivax* malaria, with and without PQ, on the risk of early *P*. *vivax* recurrence.

## Methods

### Search strategy and selection criteria

MEDLINE, Web of Science, Embase, and Cochrane Data of Systematic Reviews were searched in accordance with the Preferred Reporting Items for Systematic Reviews and Meta-Analyses (PRISMA) statement (S1 Checklist). As previously described [9,16], prospective studies evaluating the efficacy of antimalarials against uncomplicated *P*. *vivax* that were published between January 1, 2000, and January 31, 2018, in any language were identified (S1 Text).

Two independent investigators undertook the review and extracted the data (RJC and RNP), resolving discrepancy through discussion. Principal investigators of eligible studies were invited to share individual patient data and any additional data from eligible unpublished studies. Studies were included if they assessed treatment with DP or AL with or without PQ. Studies were excluded if adjunctive drugs were given or PQ was dosed weekly. Individual patient data shared with the WorldWide Antimalarial Resistance Network (WWARN) were curated and standardised as described in a data management plan [17].

Data included in this analysis were obtained in accordance with ethical approvals from the location of origin. See S2 Text for a list of specific ethics committees. Data are requested to be shared anonymised, and additional review from an ethics committee was not required for the subsequent analysis according to guidelines of the Oxford Central University Research Ethics Committee.

### Procedures

Individual patient data were excluded if baseline parasitaemia involved mixed species or blood schizontocidal treatment was incomplete. Early PQ was defined as commencement of PQ within the first 3 days of ACT treatment. Drug doses were calculated from the number of tablets a patient was administered if data were available or were based on the study protocol, assuming complete adherence. ACT partner drugs were considered determinants for recurrence rather than artemisinins, which have short half-lives and are considered determinants for parasite clearance.

Study sites were classified as having short (median time to patent relapse of 47 days or less) or long *P*. *vivax* relapse periodicity based on estimates from the Malaria Atlas Program for geographical location [12]. PQ dose was defined as a low dose if the total dose was <5.0 mg/kg or high dose if the total dose was ≥5.0 mg/kg [18]. A symptomatic recurrence was defined as recurrent parasitaemia associated with a temperature of ≥37.5°C or a history of fever within the preceding 24 hours.

Underdosing of piperaquine and lumefantrine was defined as a dose less than the minimum dose recommended by current World Health Organization (WHO) guidelines for *P*. *falciparum* [19]. DP dosing guidelines were updated by WHO in 2015, after enrolment of patients in all studies included in this analysis was completed. The revised WHO guidelines recommend a minimum total dose of piperaquine of ≥60 mg/kg in patients weighing <25 kg and ≥48 mg/kg if weighing ≥25 kg [19–21]. A minimum total dose of lumefantrine of ≥29 mg/kg is recommended in patients treated with AL [19].

### Outcomes

The primary outcome was the risk of *P*. *vivax* recurrence between days 7 and 42. The secondary outcomes were the risk of *P*. *vivax* recurrence between days 7 and 28 and days 7 and 63.

### Statistical analysis

Analyses were undertaken according to an a priori statistical analysis plan [22], unless stated otherwise, using Stata (version 15.0) and R (version 3.4.0). The protocol was registered in the International Prospective Register of Systematic Reviews (PROSPERO: CRD42016053310) prior to undertaking the analysis.

To investigate inclusion bias, baseline characteristics of targeted studies not included in the analysis were compared with the characteristics of the studies included in the analysis. Study quality and risk of bias were assessed according to criteria for individual patient data meta-analyses [23].

Kaplan-Meier survival analyses were used to calculate risk of recurrence according to the treatment administered. Patients were censored at the first day of any of the following for an outcome prior to day of the end point: recurrent parasitaemia, the last clinic visit, the last visit prior to a gap of >18 days between parasite microscopy results, the day of outcome, or the day of PQ treatment (if ≥28 days after enrolment).

The association between schizontocidal treatment (AL versus DP), coadministration of PQ, and the mg/kg dose of each drug component (piperaquine and lumefantrine) with the rate of *P*. *vivax* recurrence was estimated using Cox’s proportional hazards regression. Analyses were adjusted for potential confounders: age, sex, baseline parasitaemia, baseline haemoglobin, and regional relapse periodicity, with shared frailty applied for study site. Weight was not included, because of collinearity with age and geographical region, and parasite prevalence was not included, because of collinearity with relapse periodicity. Age was categorised for analyses of mg/kg dose as defined previously by age-related dose effects in *P*. *falciparum* [21,24]. Proportional hazards assumptions were tested using Schoenfeld residuals. Figures of risk of recurrence were generated based on the Cox model, which adjusted for confounders and assumed no study-site effect. In a sensitivity analysis, the coefficient of variation was calculated for estimates of primary analyses by removal of one study site at a time to investigate study site heterogeneity. The coefficient of variation should be interpreted in association with the absolute variation of the effect size. A subgroup analysis restricted the analysis to results from studies directly comparing DP and AL.

Following preplanned statistical analyses, a post hoc analysis was undertaken to investigate whether a delay in recurrence was associated with a benefit in clinically relevant outcomes. In patients with a *P*. *vivax* recurrence between day 7 and day 63, the delay in recurrence was correlated with the mean haemoglobin at the day of recurrence using linear regression, adjusting for confounding factors (age, sex, baseline parasitaemia, baseline haemoglobin, parasitaemia at recurrence, regional relapse periodicity, and PQ use), and with random effects for study site. Analysis was undertaken separately for all recurrences and just symptomatic recurrences.

## Results

Of 180 prospective *P*. *vivax* studies published between January 1, 2000, and January 31, 2018, 26 studies (14.4%) with patients treated with DP or AL were eligible for inclusion. Individual patient data were available from 18 studies enrolling 4,946 patients, of which 3,640 were infected with *P*. *vivax* (67.7% of the 5,377 targeted patients with *P*. *vivax*). Additional data were available for 45 patients enrolled in these published studies, but not formally reported, and 736 patients from two unpublished studies.

**Fig 1:**
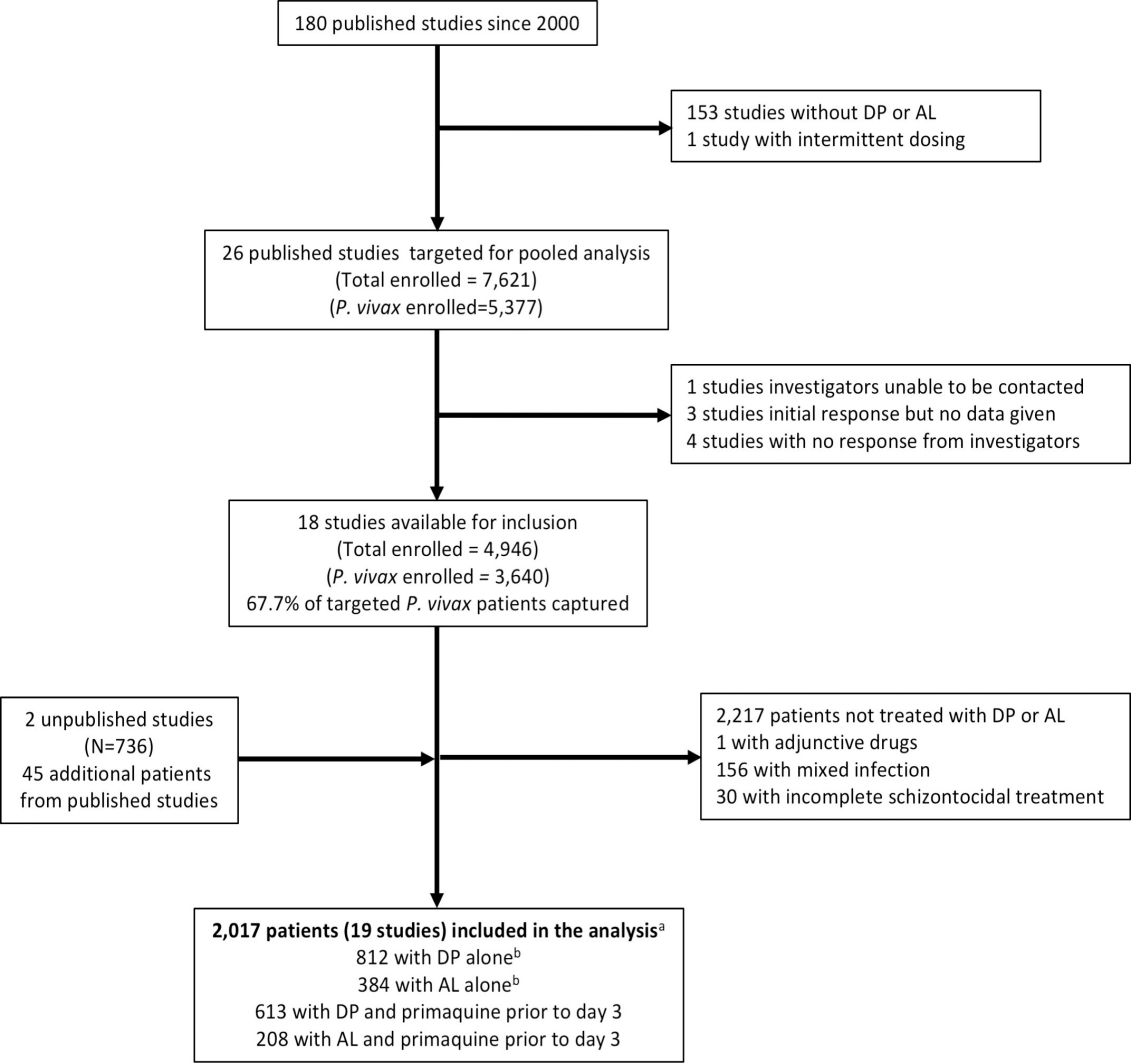
Study flowchart. ^a^One study was excluded entirely because patients treated with AL or DP all had mixed infections [43]. ^b^Includes 100 patients treated with DP and two patients treated with AL who received primaquine at day 28 and were censored at this day. AL, artemether-lumefantrine; DP, dihydroartemisinin-piperaquine.

Of 4,421 *P*. *vivax* patients with data available from eligible studies, 2,217 (50.1%) were not treated with DP or AL, and an additional 187 (4.2%) were excluded for other reasons, resulting in 2,017 patient records included in the analysis (Fig 1 and S1–S4 Tables). Of those patients, the median age was 18.0 years (interquartile range [IQR] 8.0–30.0), and 272 (13.5%) were aged <5 years (Table 1). There were 1,593 patients (79.0%) from 15 studies from the Asia-Pacific region [6,25–38], 341 patients (16.9%) from three studies from Africa [39–41], and 83 patients (4.1%) from one study from The Americas [42] (Table 1 and S1 Fig). In total, 812 patients were treated with DP alone [6,25–27,30,34,37,38], 384 with AL alone [6,26,28,31,36,39–41], 613 with DP plus early PQ [25,29,32,33,35], and 208 with AL plus early PQ [28,36,39,41,42]. Only two studies (from Indonesia and Papua New Guinea) compared treatment with DP and AL directly [6,26]. Patients were followed for 28 days in one study (*n* = 38, 1.9%) [39], 42–62 days in 10 studies (*n* = 814, 40.4%) [6,25,26,28,31,32,36–38,40], 63–84 days in three studies (*n* = 390, 19.3%) [27,34,42], and 365 days in five studies (*n* = 775, 38.4%) [29,30,33,35,41]. The mg/kg doses of lumefantrine and piperaquine were calculated from the number of tablets given for 1,281 (63.5%) of 2,017 patients, with dosing for the remaining 736 (36.5%) patients calculated from the study protocol (Table 1).

**Table 1:** Demographics and baseline characteristics.

Characteristic	DP alone	DP and early PQ	AL alone	AL and early PQ	Overall
	(*n* = 812)	(*n* = 613)	(*n* = 384)	(*n* = 208)	(*n* = 2,017)
Sex					
Female	349 (43.0%)	294 (48.0%)	144 (37.5%)	75 (36.1%)	862 (42.7%)
Male	463 (57.0%)	319 (52.0%)	240 (62.5%)	133 (63.9%)	1,155 (57.3%)
Age (years)					
Median (IQR)	16.0 (7–27.0)	21.0 (12.0–32.0)	13.0 (4.9–24.0)	28.0 (18.5–40.6)	18.0 (8.0–30.0)
<5	124 (15.3%)	42 (6.9%)	96 (25.0%)	10 (4.8%)	272 (13.5%)
5 to <15	252 (31.0%)	166 (27.1%)	115 (29.9%)	29 (13.9%)	562 (27.9%)
≥15	436 (53.7%)	405 (66.1%)	173 (45.1%)	169 (81.2%)	1,183 (58.7%)
Weight (kg)					
Median (IQR)	45.0 (18.0–54.0)	46.0 (30.0–55.0)	32.0 (15.0–55.0)	61.6 (47.0–71.5)	46.0 (20.0–56.0)
5 to <15	126 (15.5%)	40 (6.5%)	93 (24.2%)	10 (4.8%)	269 (13.3%)
15 to <25	155 (19.1%)	85 (13.9%)	69 (18.0%)	20 (9.6%)	329 (16.3%)
25 to <35	48 (5.9%)	60 (9.8%)	36 (9.4%)	8 (3.8%)	152 (7.5%)
35 to <45	76 (9.4%)	82 (13.4%)	19 (4.9%)	6 (2.9%)	183 (9.1%)
45 to <55	208 (25.6%)	182 (29.7%)	66 (17.2%)	31 (14.9%)	487 (24.1%)
55 to <80	193 (23.8%)	156 (25.4%)	99 (25.8%)	100 (48.1%)	548 (27.2%)
≥80	6 (0.7%)	8 (1.3%)	2 (0.5%)	33 (15.9%)	49 (2.4%)
Relapse periodicity					
Long	265 (32.6%)	0 (0%)	230 (59.9%)	194 (93.3%)	689 (34.2%)
Short	547 (67.4%)	613 (100.0%)	154 (40.1%)	14 (6.7%)	1,328 (65.8%)
Geographical region					
Asia-Pacific	812 (100.0%)	613 (100.0%)	154 (40.1%)	14 (6.7%)	1,593 (79.0%)
The Americas	0 (0%)	0 (0%)	0 (0%)	83 (39.9%)	83 (4.1%)
Africa	0 (0%)	0 (0%)	230 (59.9%)	111 (53.4%)	341 (16.9%)
Prevalence of *P*. *vivax*					
Low	301 (37.1%)	246 (40.1%)	223 (58.1%)	111 (53.4%)	881 (43.7%)
Moderate	61 (7.5%)	0 (0%)	28 (7.3%)	14 (6.7%)	103 (5.1%)
High	450 (55.4%)	367 (59.9%)	133 (34.6%)	83 (39.9%)	1,033 (51.2%)
Enrolment clinical variables					
Parasitaemia, parasites per μL	4,458 (1,729–9,368)	2,240 (528–6,960)	4,282 (1,672–10,480)	3,400 (1,560–7,920)	3,599 (1,200–8,784)
Haemoglobin, g/dL^a^	11.7 (2.1)	12.4 (2.0)	12.1 (2.6)	13.4 (1.9)	12.2 (2.2)
Anaemic, haemoglobin < 10 g/dL	130/764 (17.0%)	55/612 (9.0%)	72/333 (21.6%)	4/184 (2.2%)	261/1,893 (13.8%)
Gametocytes present	524/570 (91.9%)	411/411 (100%)	218/281 (77.6%)	73/175 (41.7%)	1,226/1,437 (85.3%)
Fever, temperature > 37.5°C	332/753 (44.1%)	260/556 (46.8%)	142/380 (37.4%)	58/124 (46.8%)	792/1,813 (43.7%)
Mg/kg dose calculated by tablet number	772/812 (95.1%)	112/613 (18.3%)	213/384 (55.5%)	184/208 (88.5%)	1,281/2,017 (63.5%)

Data are number (%), median (IQR), mean (SD), or *n*/*N* (%). Some percentages do not add up to 100, because of rounding.

^a^Data not available for 124 of 2,017 patients: 48 in the DP alone group, 1 in the DP plus PQ group, 51 in the AL-alone group, and 24 in the AL plus PQ group.

Abbreviations: AL, artemether-lumefantrine; DP, dihydroartemisinin-piperaquine; IQR, interquartile range; PQ, primaquine; SD, standard deviation

All patients treated with DP or DP + PQ were enrolled from the Asia-Pacific region, and those treated with DP + PQ were all from regions with short relapse periodicity. Patients treated with AL or AL + PQ were enrolled from the Asia-Pacific region, Africa, and The Americas, although patients from The Americas were all treated with AL + PQ. Compared with patients treated with DP or AL alone, patients treated with PQ were older and had lower baseline parasitaemias and a lower risk of anaemia at enrolment (Table 1). Characteristics of patients from studies that were targeted but not included were similar, although included patients were younger and had a more even balance of males and females (S5 Table). The risk of bias relating to included studies is described in S6 Table.

### Schizontocidal treatment with DP alone

Among the 812 patients treated with DP alone, the median total dose of dihydroartemisinin administered was 6.4 mg/kg (IQR 6.0–7.1; range 3.5–10.6), and the median total dose of piperaquine administered was 51.4 mg/kg (IQR 48.0–56.5; range 28.2–84.7) (S2 Fig). In total, 181 patients (22.3%) were administered less than the former WHO target dose, and 303 (37.3%) patients were administered less than the revised WHO target dose (S3 Fig).

Following DP alone, 50 of 812 patients had a recurrence between days 7 and 42, the cumulative risk of recurrence being 1.2% (95% confidence interval [CI] 0.6–2.3) at day 28 and 9.3% (95% CI 7.1–12.2) at day 42. Of the 505 patients from nine studies with follow-up beyond day 42, an additional 105 patients had a recurrence up to day 63, cumulative risk 39.9% (95% CI 33.9–46.7) at day 63. Risks for individual studies are presented in the Supporting information (S4 Fig). After adjusting for age, sex, baseline parasitaemia, baseline haemoglobin, regional relapse periodicity, and study-site clustering, an increased piperaquine dose was associated with a reduced rate of recurrence between days 7 and 42 (adjusted hazard ratio [AHR] for every 5-mg/kg increase 0.63, 95% CI 0.48–0.84, *p* = 0.0013) (Table 2). Other factors associated with *P*. *vivax* recurrence were short relapse periodicity (AHR 27.28, 95% CI 4.53–164.14, *p* < 0.001) and lower baseline haemoglobin (AHR per g/dL 0.83, 95% CI 0.68–0.99, *p* = 0.0432). When the method of calculating the dose of drug was included in the model, it was not an independent predictor of recurrence. In the sensitivity analysis, the coefficient of variation for piperaquine dose estimate was 6.08%, with AHRs ranging from 0.60 to 0.75 (S7 Table). The association between increased piperaquine dose and a reduced rate of recurrence was also apparent when the follow period was extended to day 63 (AHR for every 5-mg/kg increase 0.83, 95% CI 0.73–0.94, *p* = 0.0033).

**Table 2:** Risk factors for *P*. *vivax* recurrence between days 7 and 42 in patients treated with dihydroartemisinin-piperaquine alone.

Risk factor	Total *N* (*n*)^a^	Adjusted HR (95% CI)	*p*-Value
Piperaquine dose, every 5-mg/kg increase	764 (41)	0.63 (0.48–0.84)	0.0013
Age, years			
≥15	432 (21)	Reference	-
<5	82 (14)	1.69 (0.68–4.23)	0.2615
5 to <15	250 (6)	0.70 (0.27–1.85)	0.4741
Gender			
Male	441 (31)	Reference	-
Female	323 (10)	0.64 (0.31–1.32)	0.2231
Enrolment clinical variables			
Parasitaemia, parasites per μL every 10-fold increase	764 (41)	1.19 (0.73–1.93)	0.4818
Haemoglobin, every 1-g/dL increase	764 (41)	0.83 (0.68–0.99)	0.0432
Relapse periodicity			
Long	264 (2)	Reference	-
Short	500 (39)	27.28 (4.53–164.14)	<0.001

Theta (variance of frailty parameter for clustering of study sites) = 0.41. The assumption of proportional hazards held for the model (*p* = 0.20 for global test). To examine the robustness of the parameter estimates, a sensitivity analysis was carried out by removing one study site at a time, which showed that the overall coefficient of variation for piperaquine dose estimates in the multivariable model was small (S7 Table).

^a^Number of patients (number with recurrence by day 42).

Abbreviations: CI, confidence interval; HR, hazard ratio

To investigate the current WHO guidelines for DP dosing, piperaquine dosing was dichotomised into a dose above and below the minimum WHO-recommended dose for *P*. *falciparum* in the model. A piperaquine dose under the minimum WHO-recommended dose was associated with an increased rate of recurrence by day 42 (AHR 2.49, 95% CI 1.19–5.22, *p* = 0.0159). By day 63, the association between piperaquine dose and the rate of recurrence was attenuated (AHR 1.47, 95% CI 0.96–2.26, *p* = 0.0797), although it remained significant in children weighing <25 kg (AHR 2.33, 95% CI 1.18–4.62, *p* = 0.0149).

### Schizontocidal treatment with AL alone

A total of 384 patients were treated with AL alone. The median total dose administered was 10.0 mg/kg (IQR 8.6–12.0; range 4.6–20.0) of artemether and 60.0 mg/kg (IQR 51.4–72.0; range 27.7–120.0) of lumefantrine (S5 Fig and S6 Fig).

Following AL alone, 136 of 395 patients had a recurrence between days 7 and 42, with a cumulative risk of recurrence of 21.0% (95% CI 16.9–25.8) at day 28 and 44.0% (95% CI 38.7–49.8) at day 42. Of 14 patients with follow-up beyond day 42 and up to day 63, 11 (78.6%) had recurrent parasitaemia. Only one patient (0.3%) was administered less than the current dose of lumefantrine recommended by WHO for *P*. *falciparum* (<29 mg/kg), and this patient did not have a recurrent infection (S6 Fig). The median dose of lumefantrine was 65.5 mg/kg in patients younger than 5 years and 53.3 mg/kg in those older than 5 years. After adjusting for confounders and study-site clustering, there was no significant effect of lumefantrine dose on rate of *P*. *vivax* recurrence (AHR per 5 mg/kg 1.07, 95% CI 0.99–1.16, *p* = 0.0869). Low baseline haemoglobin was a risk factor for recurrence (Table 3).

**Table 3:** Risk factors for *P*. *vivax* recurrence between days 7 and 42 in patients treated with artemether-lumefantrine alone.

Risk factor	Total *N* (*n*)^a^	Adjusted HR (95% CI)	*p*-Value
Lumefantrine dose, every 5-mg/kg increase	333 (119)	1.07 (0.99–1.16)	0.0869
Age, years			
≥15	171 (37)	Reference	-
<5	59 (33)	1.41 (0.78–2.58)	0.2570
5 to <15	103 (49)	1.27 (0.71–2.27)	0.4110
Gender			
Male	212 (69)	Reference	-
Female	121 (50)	0.85 (0.58–1.24)	0.3982
Enrolment clinical variables			
Parasitaemia, parasites per μL every 10-fold increase	333 (119)	1.35 (0.96–1.89)	0.3982
Haemoglobin, every 1-g/dL increase	333 (119)	0.88 (0.80–0.96)	0.0862
Relapse periodicity			
Long	216 (75)	Reference	-
Short	117 (44)	1.31 (0.79–2.15)	0.2917

Theta (variance of frailty parameter for clustering of study sites) = 0.01. The assumption of proportional hazards held for the model (*p* = 0.33 for global test).

^a^Number of patients (number with recurrence by day 42).

Abbreviations: CI, confidence interval; HR, hazard ratio

### Schizontocidal treatment AL versus DP

**Fig 2:**
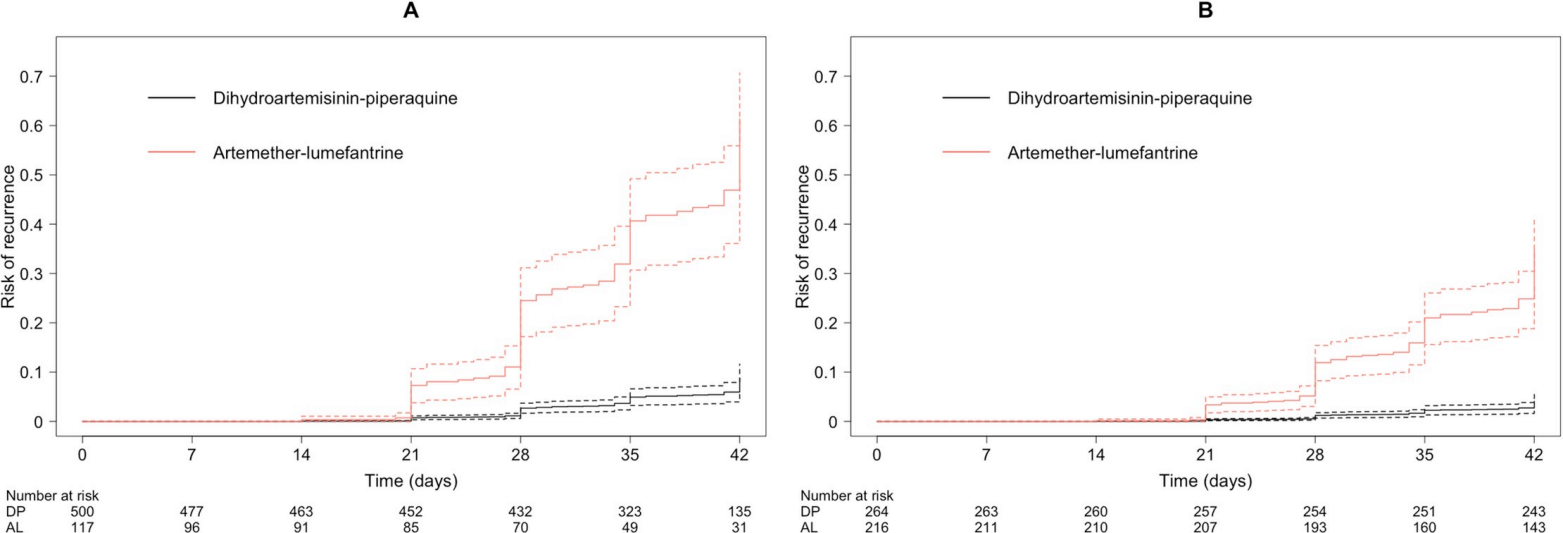
**Risk of recurrence (solid line, estimate; dashed lines, limits of 95% CI) adjusted for age, gender, relapse periodicity, baseline haemoglobin, and baseline parasitaemia in patients receiving DP or AL alone in (A) short-periodicity regions (*p* < 0.001) and (B) long-periodicity regions (*p* < 0.001).** Assumes zero effect from study site; *p*-values derived from Cox model. AL, artemether-lumefantrine; CI, confidence interval; DP, dihydroartemisinin-piperaquine.

In the subgroup of patients not treated with PQ, the rate of *P*. *vivax* recurrence between days 7 and 42 was significantly greater following AL than DP after adjusting for confounders and study-site clustering (AHR 12.63, 95% CI 6.40–24.92, *p* < 0.001) (Table 4). Because of violation of the proportional hazards assumption, the analysis was undertaken separately, stratifying by relapse periodicity. The high rate of recurrence after AL was apparent both in regions of short relapse periodicity (AHR 6.56, 95% CI 4.04–10.65, *p* < 0.001) and long relapse periodicity (AHR 81.40, 95% CI 19.58–338.45, *p* < 0.001) (Fig 2 and Table 4). In the sensitivity analysis, there was minimal bias related to individual study sites (S8 Table). The difference between DP and AL was also apparent in a subgroup analysis of the two studies comparing the treatments directly (AHR 10.27, 95% CI 3.99–26.45, *p* < 0.001). The method of drug dose calculation was not an independent predictor of recurrence when included in the overall model (AHR 0.57 [95% CI 0.19–1.70] for dosing based on the actual number of tablets administered compared with that expected from study protocol; *p* = 0.3137).

**Table 4:** Multivariable model for rate of *P*. *vivax* recurrence between days 7 and 42 in patients receiving artemether-lumefantrine or dihydroartemisinin-piperaquine alone.

Risk factor	All regions			Long relapse periodicity			Short relapse periodicity		
	Total *N* (*n*)^a^	Adjusted HR (95% CI)	*p*-Value	Total *N* (*n*)^a^	Adjusted HR (95% CI)	*p*-Value	Total *N* (*n*)^a^	Adjusted HR (95% CI)	*p*-Value
Treatment									
Dihydroartemisinin-piperaquine	764 (41)	Reference	-	264 (2)	Reference	-	500 (39)	Reference	-
Artemether-lumefantrine	333 (119)	12.63 (6.40–24.92)	<0.001	216 (75)	81.40 (19.58–338.45)	<0.001	117 (44)	6.56 (4.04–10.65)	<0.001
Age, per every 5-year increase	1,097 (160)	0.93 (0.87–1.00)	0.0562	480 (77)	0.88 (0.78–1.00)	0.0521	617 (83)	1.00 (0.91–1.09)	0.9153
Gender									
Male	653 (100)	Reference	-	271 (45)	Reference	-	382 (55)	Reference	-
Female	444 (60)	0.74 (0.53–1.04)	0.0840	209 (32)	1.12 (0.70–1.79)	0.6269	235 (28)	0.47 (0.29–0.76)	0.0021
Parasitaemia, parasites per μL every 10-fold increase	1,097 (160)	1.44 (1.09–1.91)	0.0107	480 (77)	1.43 (0.86–2.38)	0.1700	617 (83)	1.37 (0.99–1.89)	0.0600
Baseline haemoglobin, every 1-g/dL increase	1,097 (160)	0.87 (0.80–0.94)	<0.001	480 (77)	0.84 (0.74–0.94)	0.0030	617 (83)	0.86 (0.78–0.96)	0.0053
Relapse periodicity									
Long	480 (77)	Reference	-	480 (77)	-	-	-	-	-
Short	617 (83)	2.95 (1.26–6.91)	0.0128	-	-	-	617 (83)	-	-

Theta (variance of frailty parameter for clustering of study sites for the model of all regions) = 0.35. The assumption of proportional hazards did not hold for the model of all regions (*p* < 0.001 according to the global test, with *p* < 0.001 for relapse periodicity and *p* < 0.001 for treatment). There were interactions between treatment and relapse periodicity and relapse periodicity and gender. For the model of patients from regions of long relapse periodicity alone, the assumption of proportional hazards held (*p* = 0.19 according to the global test). For the model of patients from regions of short relapse periodicity alone, the AHR for treatment (artemether-lumefantrine versus dihydroartemisinin-piperaquine) varied with time, with a higher AHR in the early follow-up to day 28 (AHR 18.48, 95% CI 7.32–46.69, *p* < 0.001) compared with later follow-up after day 28 (AHR 4.11, 95% CI 2.29–7.38, *p* < 0.001, respectively), consistent with the difference in elimination half-lives between lumefantrine and piperaquine. To examine the robustness of the parameter estimates, a sensitivity analysis was carried out by removing one study site at a time, which showed that the overall coefficient of variation of parameter estimates in the multivariable models was minimal (S8 Table).

^a^Number of patients (number with recurrence by day 42).

Abbreviations: AHR, adjusted HR; CI, confidence interval; HR, hazard ratio

### Schizontocidal treatment plus PQ radical cure regimens

The median dose of PQ was 6.6 mg/kg (IQR 4.7–7.0; range 2.6–8.9) in patients treated with DP and 3.3 mg/kg (IQR 3.1–3.8; range 0.3–9.1) in patients treated with AL. Of the patients treated with DP + PQ, 173 (28.2%) received a low-dose PQ regimen (<5 mg/kg), and 440 (71.8%) received a high-dose regimen (≥5.0 mg/kg). The corresponding figures for AL + PQ were 191 (91.8%) and 17 (8.2%).

There were three recurrences between days 7 and 42 in patients treated with DP + PQ, with a cumulative risk of recurrence of 0.2% (95% CI 0.0–1.2) at day 28 and 1.0% (95% CI 0.3–3.2) at day 42. Of the 55 patients with follow-up beyond day 42, a further two patients had a recurrence between days 42 and 63: cumulative risk 5.2% (95% CI 1.7–15.5) at day 63. There were nine recurrences between days 7 and 42 in patients treated with AL + PQ, with a cumulative risk of recurrence of 2.7% (95% CI 1.1–6.3) at day 28 and 5.3% (95% CI 2.8–10.0) at day 42. Two of five patients followed beyond day 42 had a recurrence between days 42 and 63.

**Fig 3:**
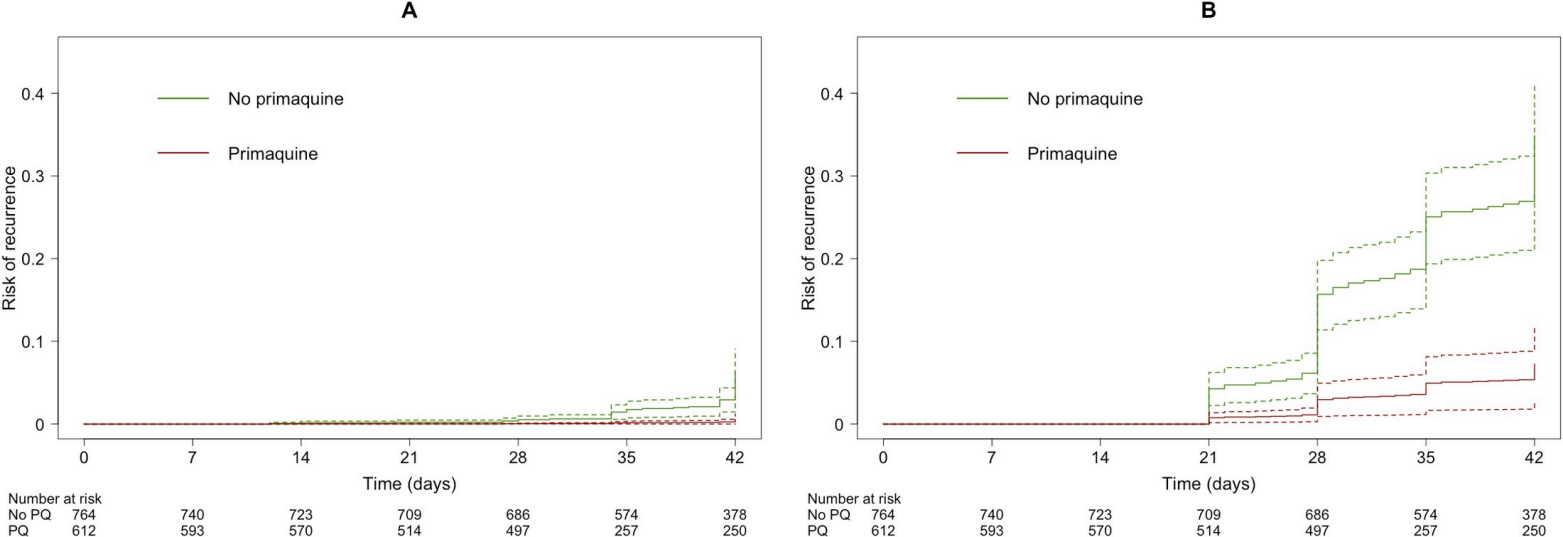
**Risk of recurrence (solid line, estimate; dashed lines, limits of 95% CI) adjusted for age, mg/kg piperaquine or lumefantrine dose, gender, relapse periodicity, baseline haemoglobin, and baseline parasitaemia comparing treatment with and without PQ in patients receiving (A) dihydroartemisinin-piperaquine (*p* = 0.0933) or (B) artemether-lumefantrine (*p* < 0.001).** Assumes zero effect from study site; *p*-values derived from Cox model. CI, confidence interval; PQ, primaquine.

In a multivariable model of patients treated with DP, recurrence by day 42 was not reduced significantly by coadministration of PQ (AHR = 0.23, 95% CI 0.04–1.28, *p* = 0.0933). However, in patients treated with AL, coadministration of PQ reduced the rate of recurrence (AHR = 0.20, 95% CI 0.10–0.41, *p* < 0.001) (Fig 3 and S9 Table). When PQ dose was included in the model of patients treated with DP, neither low-dose PQ (AHR = 0.28, 95% CI 0.04–1.99, *p* = 0.2031) nor high-dose PQ (AHR = 0.15, 95% CI 0.01–1.96, *p* = 0.1494) was associated with reduced rate of recurrence. In patients treated with AL, low-dose PQ reduced the rate of recurrence (AHR = 0.21, 95% CI 0.10–0.41, *p* < 0.001; of note, only 17 patients received high-dose PQ) (S10 Table). In a subset analysis including five studies following patients to day 63 or more, coadministration of any dose of PQ with DP reduced the rate of recurrence (AHR = 0.06, 95% CI 0.01–0.63, *p* = 0.0196) (S11 Table). In a sensitivity analysis, reductions in rate of recurrence with PQ were unlikely to be attributable to bias from individual study sites (S12 Table). Because of the low number of recurrences, a multivariable analysis comparing the risk of recurrence with DP + PQ and AL + PQ could not be done.

### Time to *P*. *vivax* recurrence and haematological recovery

In total, of the 160 patients with recurrence between day 7 and day 63 treated with DP with or without PQ, 12 had available data on the haemoglobin at the day of recurrence, 6 (50.0%) of which were symptomatic recurrences. The corresponding numbers for patients treated with AL were 109 patients with available data out of 158 patients with recurrence between day 7 and day 63, with 70 (64.2%) of these patients having symptomatic recurrence.

Because of the low number of recurrences after DP +/− PQ, a multivariable analysis assessing the effect of day of recurrence on haemoglobin could not be undertaken. For patients treated with AL +/− PQ, there was a nonsignificant increase in haemoglobin of 0.07 g/dL (95% CI −0.06 to 0.20), *p* = 0.3130 for every 5 days that recurrence was delayed. When the analysis was restricted to symptomatic recurrences, the mean haemoglobin at recurrence increased 0.13 g/dL (95% CI 0.01–0.26) for every 5 days that recurrence was delayed (*p* = 0.0407) (S13 Table).

## Discussion

In response to emerging resistance of *P*. *vivax* to chloroquine and the pragmatic advantages of a universal treatment policy, some countries are recommending ACTs for all species of malaria. Nevertheless, the optimal ACT for *P*. *vivax* remains unclear [10,11]. The pattern of relapse following antimalarial treatment is determined by the elimination kinetics of the schizontocidal treatment. In this meta-analysis of data from more than 2,000 individual patients, the risk of early vivax recurrence up to day 42 was 12.6-fold higher following AL than DP. This difference was apparent despite 37.3% of patients receiving DP doses below those recommended for *P*. *falciparum*, with these patients having a 2.5-fold increased rate of recurrence compared with those receiving a higher dose. However, by day 63, the risk of recurrence was greater than 39.9% following treatment with either ACT. This suggests that the prolonged half-life of piperaquine in DP may have only been delaying the time to the first patent relapse, as reported previously in comparisons of rapidly and slowly eliminated antimalarial drugs (quinine and mepacrine, artesunate and chloroquine) [2,44]. Coadministration of PQ was associated with the greatest reduction in the risk of *P*. *vivax* recurrence, with an 80% reduction following AL at day 42 and a 92% reduction following DP at day 63.

ACT is the recommended treatment for *P*. *falciparum* asexual stages [19]. Lumefantrine, piperaquine, and mefloquine all have high antimalarial activity against *P*. *vivax* [26,45–48]; however, they differ markedly in their pharmacokinetic profiles. ACTs achieve their antimalarial effect through an initial rapid reduction in parasite biomass attributable to the short-acting but potent artemisinin derivative, with the subsequent removal of the remaining parasites by the intrinsically less active but more slowly eliminated lumefantrine (terminal elimination half-life of approximately 4 days) or piperaquine (terminal elimination half-life of 28–35 days) [49–51]. After eradication of the asexual stages of the parasite from the peripheral blood, patients who remain in an endemic area are at risk of reinfection or, following *P*. *vivax*, from relapses from the liver-stage hypnozoites. Slowly eliminated ACT partner drugs exert a longer post-treatment prophylactic effect than more rapidly eliminated partner drugs, and this is reflected in both the rates and timing of reinfection and relapse [10]. The greater the risk of *P*. *vivax* relapse or reinfection with either species, the more apparent is this prophylactic effect. In our analysis, 70% of patients were followed for 42 days or less, and only 5% of patients were followed beyond 63 days, preventing a comprehensive assessment of the comparative efficacies of DP and AL on later recurrences. Hence, we were unable to determine whether the lower rate of recurrence following DP was the result of a reduction in the total number of relapses or merely due to a delay in the first relapse with a similar total number of relapses occurring after blood drug concentrations fall below the minimum inhibitory concentration.

Following a large meta-analysis of patients with *P*. *falciparum* and confirmatory pharmacokinetic–pharmacodynamic analysis [20,21], WHO recommended that the total dose of piperaquine in patients with *P*. *falciparum* be increased from a lower cutoff of ≥48 mg/kg in all ages to ≥60 mg/kg in children weighing <25 kg [19]. As yet, there are no guidelines on ACT dosing specifically for *P*. *vivax*, and thus, recommended doses are extrapolated from those derived for *P*. *falciparum* [19]. To our knowledge, our analysis provides the first large-scale dosing assessment of AL and DP in patients with *P*. *vivax*. Only one patient treated with AL alone was given a dose less than that recommended for *P*. *falciparum*, and there was no evidence that dosing should be different in *P*. *vivax*. In contrast, almost 40% of patients treated with DP were underdosed with piperaquine compared with the updated dosing guidelines for *P*. *falciparum*. At day 42, these patients were at 2.5-fold higher risk of recurrence compared with patients prescribed the dose recommended for *P*. *falciparum*. The effect of piperaquine dose was still apparent at day 63, although this only reached statistical significance in patients < 25 kg, who had a 2.3-fold higher risk of recurrence when underdosed. These results highlight the need for patient-centred dosing to ensure individual patients receive an appropriate dose based on body weight.

Although PQ has been used in conjunction with chloroquine for over 60 years, its combination with ACTs has been used only recently. Lumefantrine is an inhibitor of cytochrome P450 (CYP)2D6 in vitro [52,53], an enzyme considered responsible for biotransformation of PQ to its active metabolites, and this has led to concerns that coadministration will reduce the efficacy of PQ [54]. PQ pharmacokinetics are related to CYP2D6 activity, regardless of whether patients are treated with AL plus PQ, suggesting that any inhibition of CYP2D6 by lumefantrine is incomplete [55]. The current study is reassuring, demonstrating that coadministration of PQ and AL results in an 80% reduction in the rate of recurrence by day 42. Although reduction in *P*. *vivax* following coadministration of DP and PQ did not reach statistical significance at day 42, this was likely because of the relatively few recurrences at this time following DP alone, because by day 63, the risk of recurrence was reduced by over 90%.

Our study has a number of limitations. Only 68% of potentially eligible patient records were included in the analysis. There were some minor differences in age and sex between included and targeted patient records; however, included studies were more recent and included a more balanced sex distribution. Data for mg/kg dose were based on the study protocol rather than the number of tablets received in about one-third of patients. However, the method of dose calculation was not a significant predictor of recurrence, and over 95% of patients treated with DP alone had complete dosing data available, suggesting this is unlikely to have impacted upon our analysis.

Analysis was restricted to patients with follow-up to day 42 or less for AL, but in 62% of patients treated with DP, follow-up was available up to day 63. Comparison of recurrences between two drugs’ regimens with markedly different pharmacokinetic profiles exerting different post-treatment prophylaxis within this time period therefore needs to be interpreted with caution. Furthermore, admixture of new infections with relapses will be different with the two drugs, and recrudescence, relapse, and reinfection were not separated in these studies. In a post hoc analysis, a delay in the time to recurrence in patients treated with AL resulted in a higher mean haemoglobin at the time of recurrence. Hence, even if DP is delaying rather than preventing relapses, it likely offers a greater time for haematological recovery between the initial infection and the next recurrence and, thus, potential clinical benefits. Additional studies are warranted to quantify the longer-term benefits of prolonged parasite prophylaxis on the number of total relapses and cumulative risk of anaemia. Importantly, only two of the studies included in our analysis compared AL and DP directly, and none of the studies compared AL + PQ and DP + PQ; thus, treatment was not randomised, potentially confounding the interpretation of the comparative efficacies and the effect of relapse periodicity. However, reassuringly, when the analysis was restricted to these two studies, the results remained consistent.

In summary, these findings suggest that early recurrence of *P*. *vivax* parasitaemia can be reduced significantly in patients treated with DP compared with AL. Despite the relatively low risk of early recurrence with DP, over one-third of patients received a dose less than that recommended in the current *P*. *falciparum* treatment guidelines, and this was associated with an increased risk of *P*. *vivax* recurrence. Although by day 63 there was a high risk of *P*. *vivax* recurrence after both AL and DP, coadministration with PQ reduced this substantially. Our findings support recommendations that ACTs should be combined with PQ and suggest that in patients in which PQ is contraindicated, DP may provide early clinical benefits over AL alone.

### Ethics approval

Data included in this analysis were obtained in accordance with ethical approvals from the location of origin. See S2 Text for a list of specific ethics committees. Data are requested to be shared anonymised, and additional review from an ethics committee was not required for the subsequent analysis according to guidelines of the Oxford Central University Research Ethics Committee.

## Supporting information

S1 Checklist PRISMA-IPD checklist of items to include when reporting a systematic review and meta-analysis of individual participant data. PRISMA-IPD, Preferred Reporting Items for Systematic Reviews and Meta-Analyses–Individual Patient Data.
(PDF)

S1 Text Search strategy. (PDF)

S2 Text Ethics approval. (PDF)

S1 Fig Study sites for clinical trials. (PDF)

S2 Fig Histogram of drug dosing for (A) dihydroartemisinin in patients receiving dihydroartemisinin-piperaquine alone (*n* = 812), (B) piperaquine in patients receiving dihydroartemisinin-piperaquine alone (*n* = 812), (C) dihydroartemisinin in patients receiving dihydroartemisinin-piperaquine plus early primaquine (*n* = 613), (D) piperaquine in patients receiving dihydroartemisinin-piperaquine plus early primaquine (*n* = 613), and (E) primaquine in patients receiving dihydroartemisinin-piperaquine plus early primaquine (*n* = 613).
(PDF)

S3 Fig Mg/kg total drug dosing of piperaquine by bodyweight in patients receiving dihydroartemisinin-piperaquine alone (*n* = 812). (PDF)

S4 Fig Risk of recurrence (derived from complement of Kaplan-Meier estimate) by study at days (A) 28, (B) 42, and (C) 63 in patients receiving dihydroartemisinin-piperaquine alone.
(PDF)

S5 Fig Histogram of drug dosing for (A) artemether in patients receiving artemether-lumefantrine alone (*n* = 384), (B) lumefantrine in patients receiving artemether-lumefantrine alone (*n* = 384), (C) artemether in patients receiving artemether-lumefantrine plus early primaquine (*n* = 208), (D) lumefantrine in patients receiving artemether-lumefantrine plus early primaquine (*n* = 208), and (E) primaquine in patients receiving artemether-lumefantrine plus early primaquine (*n* = 208).
(PDF)

S6 Fig Mg/kg total drug dosing of lumefantrine in patients receiving artemether-lumefantrine alone (*n* = 384). (PDF)

S7 Fig Risk of recurrence by study at days 28 and 42 in patients receiving artemether-lumefantrine alone. (PDF)

S1 Table Reasons for studies not being included in analysis. (PDF)

S2 Table Studies included in analysis. (PDF)

S3 Table Study sites included in analysis. (PDF)

S4 Table Studies targeted for the analysis but not included. (PDF)

S5 Table Comparison of baseline characteristics between included and targeted studies. (PDF)

S6 Table Risk of bias assessment. (PDF)

S7 Table Sensitivity analysis investigating the effect of piperaquine dose on the rate of *P. vivax* recurrence between days 7 and 42 for patients that received dihydroartemisinin-piperaquine alone. (PDF)

S8 Table Sensitivity analysis investigating the effect of schizontocidal treatment on the rate of *P. vivax* recurrence between days 7 and 42 for patients that received dihydroartemisinin-piperaquine or artemether-lumefantrine alone. (PDF)

S9 Table Multivariable models for effect of primaquine use on the rate of *P. vivax* recurrence between days 7 and 42 in patients receiving dihydroartemisinin-piperaquine or artemether-lumefantrine. (PDF)

S10 Table Multivariable models for effect of primaquine dose on the rate of *P. vivax* recurrence between days 7 and 42 in patients receiving dihydroartemisinin-piperaquine or artemether-lumefantrine. (PDF)

S11 Table Multivariable models for effect of primaquine on the rate of *P. vivax* recurrence between days 7 and 63 in patients receiving dihydroartemisinin-piperaquine. (PDF)

S12 Table Sensitivity analysis investigating the effect of primaquine use on the rate of *P. vivax* recurrence between days 7 and 42. (PDF)

S13 Table Multivariable models for effect of day of recurrence on the haemoglobin at recurrence in patients treated with artemether-lumefantrine with or without primaquine. (PDF)

S1 References References for studies identified in the systematic review but not included in the analysis. (PDF)

## Abbreviations

ACT: artemisinin-based combination therapy
AHR: adjusted hazard ratio
AL: artemether-lumefantrine
CI: confidence interval
CYP: cytochrome P450
DP: dihydroartemisinin-piperaquine
HR: hazard ratio
IQR: interquartile range
PRISMA: Preferred Reporting Items for Systematic Reviews and Meta-Analyses
PROSPERO: International Prospective Register of Systematic Reviews
PQ: primaquine
SD: standard deviation
WHO: World Health Organization
WWARN: WorldWide Antimalarial Resistance Network
