# Supplementary material for: The efficacy of dihydroartemisinin-piperaquine and artemether-lumefantrine with and without primaquine on Plasmodium vivax recurrence: A systematic review and individual patient data meta-analysis
Source: PLoS Med. 2019 Oct 4;16(10):e1002928. doi: 10.1371/journal.pmed.1002928 (PMC6777759; doi:10.1371/journal.pmed.1002928)
Supplement: S1 Text — (PDF) [file pmed.1002928.s002.pdf]

S1 Text. Search strategy

Search strategy

All prospective *P. vivax* antimalarial clinical trials with a minimum of 28 days follow up, published between Jan 1, 2000 and Jan 31, 2018 were identified by the application of the key terms (listed below) through Medline (Pubmed), Web of Science, Embase and the Cochrane Database of Systematic Reviews. Abstracts of all references containing any mention of antimalarial drugs were manually checked to confirm prospective clinical trials, with review of full text when needed. Studies on prevention, prophylaxis, reviews, animal studies, patients with severe malaria, where schizontocidal treatment was unsupervised or where data were extracted retrospectively from medical records outside of a planned trial were excluded. The review process was undertaken by two independent investigators who also performed data extraction (RJC and RNP), and is documented in more detail in Commons *et al*, Int J Parasitol Drug Drug Res 2017 [16] and Commons *et al*, Lancet Infect Dis 2018 [9].

Key terms

Literature search (conducted January 2018) with the following key terms (version undertaken in Pubmed): (malaria OR plasmod*) AND (amodiaquine OR atovaquone OR artemisinin OR arteether OR artesunate OR artemether OR artemether OR artemotil OR azithromycin OR artekin OR chloroquine OR chlorproguanil OR cycloguanil OR clindamycin OR coartem OR dapsone OR dihydroartemisinin OR duo-cotecxin OR doxycycline OR halofantrine OR lumefantrine OR lariam OR malarone OR mefloquine OR naphthoquine OR naphthoquinone OR piperaquine OR primaquine OR proguanil OR pyrimethamine OR pyronaridine OR proguanil OR quinidine OR quinine OR riamet OR sulphadoxine OR tetracycline OR tafenoquine).
