# Supplementary material for: The efficacy of dihydroartemisinin-piperaquine and artemether-lumefantrine with and without primaquine on Plasmodium vivax recurrence: A systematic review and individual patient data meta-analysis
Source: PLoS Med. 2019 Oct 4;16(10):e1002928. doi: 10.1371/journal.pmed.1002928 (PMC6777759; doi:10.1371/journal.pmed.1002928)
Supplement: S2 Text — (PDF) [file pmed.1002928.s003.pdf]

S2 Text. Ethics approval

Data included in this analysis were obtained in accordance with ethical approvals from the location of origin. Specific approvals for each study were granted as follows: *Hasugian-2007*, National Institute of Health Research and Development, Indonesian Ministry of Health, Jakarta, Indonesia, and Menzies School of Health Research, Darwin, Australia; *Ratcliff-2007*, National Institute of Health Research and Development, Indonesian Ministry of Health, Jakarta, Indonesia, Menzies School of Health Research, Darwin, Australia, and the Oxford Tropical Research Ethics Committee, Oxford, UK; *Karunajeewa-2008*, Papua New Guinea Ministry of Health Medical Research Advisory Committee, and the University of Western Australia Human Research Ethics Committee; *Awab-2010*, Faculty of Tropical Medicine, Mahidol University, Thailand, the Oxford Tropical Research Ethics Committee, Oxford University, UK and the Institutional Review Board of the Afghan Public Health Institute, Ministry of Public Health, Afghanistan; *Phyo-2011*, the Faculty of Tropical Medicine, Mahidol University and the Oxford Tropical Research Ethics Committee, Oxford University; *Abdallah-2012*, Health Research Board at the Ministry of Health in Kassala state, Eastern Sudan; *Barber-2013*, Ethics Committees of the Malaysian Ministry of Health and Menzies School of Health Research; *Hwang-2013*, U.S. Centers for Disease Control and Prevention, Columbia University, and the Ethiopian Public Health Association; *Pasaribu-2013*, the National Institute of Health Research and Development, Indonesian Ministry of Health, Jakarta, Indonesia, Faculty of Tropical Medicine, Mahidol University, Thailand, and the Oxford Tropical Research Ethics Committee, Oxford University, United Kingdom; *Sutanto-2013*, Ethical Committee of the Faculty of Medicine, University of Indonesia, Jakarta; *Laman-2014*, PNG Institute of Medical Research Review Board, the Medical Research Advisory Committee of PNG, and the University of Western Australia Human Research Ethics Committee; *Lidia-2015*, the Faculty of Medicine of Gadjah Mada University, Indonesia; *Nelwan-2015*, the Faculty of Medicine, University of Indonesia and the Centre for Tropical Medicine, Nuffield Department of Medicine, University of Oxford; *Thuan-2016*, the Ho Chi Minh Institute of Malaria-Parasitology-Entomology and the Oxford University Tropical Research Ethics Board; *Abreha-2017*, National Research Ethics Review Committee in Ethiopia, Menzies School of Health Research, Darwin, Australia, the US Centers for Disease Control and Prevention Institutional Review Board Board, and the Columbia University Institutional Review Board; *Chu-2018*, Mahidol University Faculty of Tropical Medicine Ethics Committee and the Oxford Tropical Research Ethics Committee; *Grigg-2018*, Ministry of Health, Malaysia, London School of Hygiene and Tropical Medicine, United Kingdom, and Menzies School of Health Research, Australia; *Daher-2018*, the Tropical Medicine Research Center of Rondonia (CEPEM), Porto Velho, Brazil; *Poespoprodjo-2018*, Eijkman Institute Research Ethics Commission, Jakarta, Indonesia and Menzies School of Health Research, Darwin, Australia. Data are anonymised and review from an ethics committee was not required according to guidelines of the Oxford Central University Research Ethics Committee.
