# Supplementary material for: The efficacy of dihydroartemisinin-piperaquine and artemether-lumefantrine with and without primaquine on Plasmodium vivax recurrence: A systematic review and individual patient data meta-analysis
Source: PLoS Med. 2019 Oct 4;16(10):e1002928. doi: 10.1371/journal.pmed.1002928 (PMC6777759; doi:10.1371/journal.pmed.1002928)
Supplement: S2 Fig — Histogram of drug dosing for (A) dihydroartemisinin in patients receiving dihydroartemisinin-piperaquine alone (n = 812), (B) piperaquine in patients receiving dihydroartemisinin-piperaquine alone (n = 812), (C) dihydroartemisinin in patients receiving dihydroartemisinin-piperaquine plus early primaquine (n = 613), (D) piperaquine in patients receiving dihydroartemisinin-piperaquine plus early primaquine (n = 613), and (E) primaquine in patients receiving dihydroartemisinin-piperaquine plus early primaquine (n = 613). (PDF) [file pmed.1002928.s005.pdf]

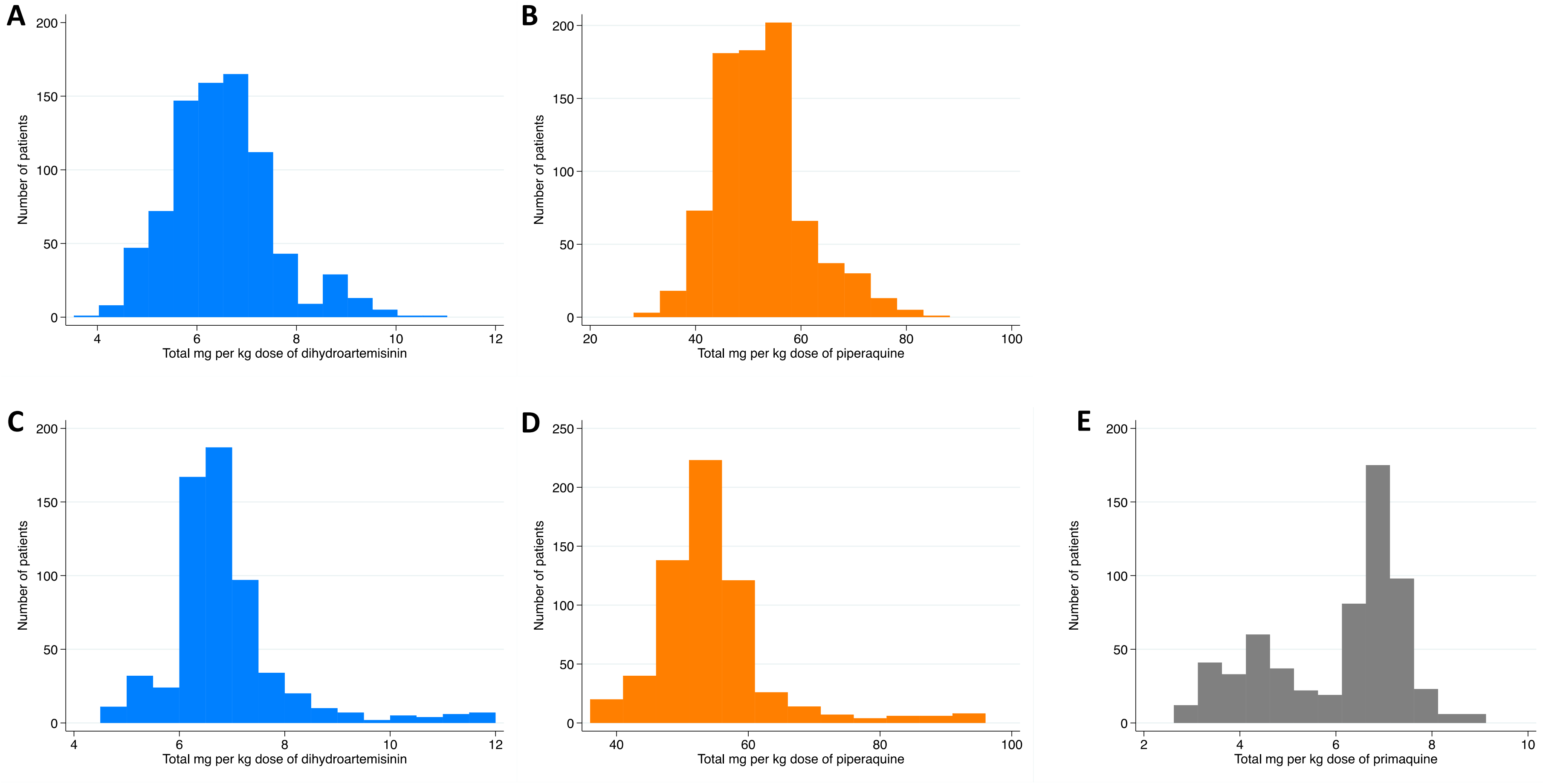

S2 Fig. Histogram of drug dosing for (A) dihydroartemisinin in patients receiving dihydroartemisinin-piperaquine alone (n=812), (B) piperaquine in patients receiving dihydroartemisinin-piperaquine alone (n=812), (C) dihydroartemisinin in patients receiving dihydroartemisinin-piperaquine plus early primaquine (n=613), (D) piperaquine in patients receiving dihydroartemisinin-piperaquine plus early primaquine (n=613) and (E) primaquine in patients receiving dihydroartemisinin-piperaquine plus early primaquine (n=613).
