# Supplementary material for: The efficacy of dihydroartemisinin-piperaquine and artemether-lumefantrine with and without primaquine on Plasmodium vivax recurrence: A systematic review and individual patient data meta-analysis
Source: PLoS Med. 2019 Oct 4;16(10):e1002928. doi: 10.1371/journal.pmed.1002928 (PMC6777759; doi:10.1371/journal.pmed.1002928)
Supplement: S3 Fig — (PDF) [file pmed.1002928.s006.pdf]

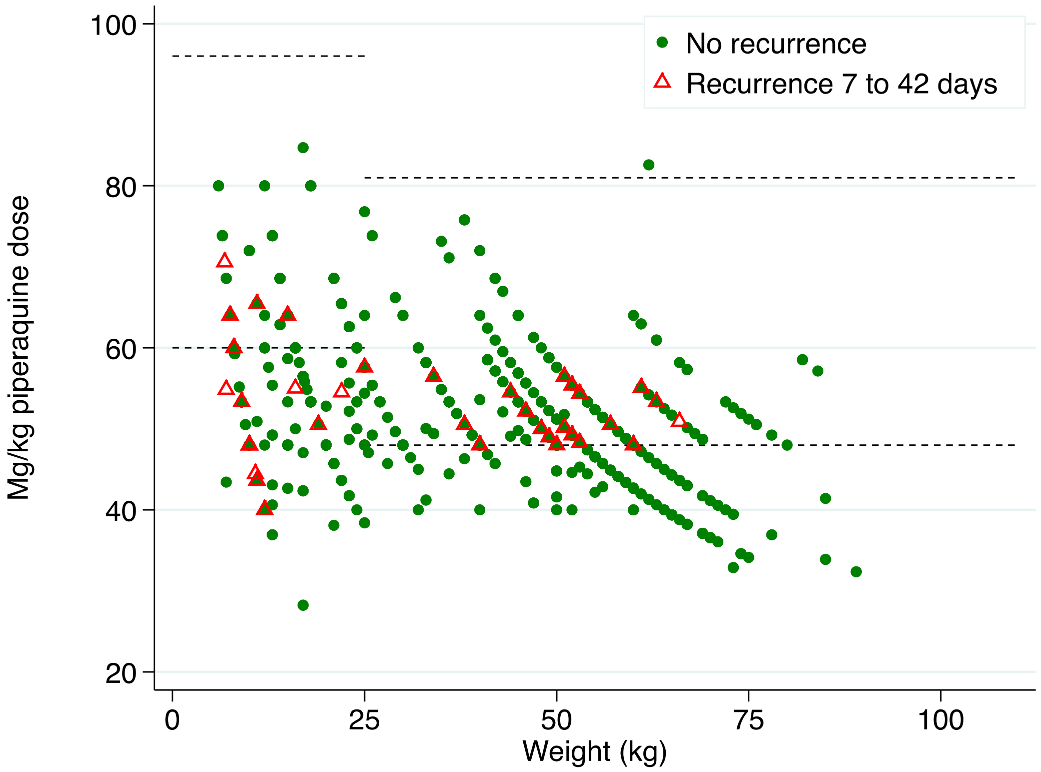

S3 Fig. Mg/kg total drug dosing of piperaquine by bodyweight in patients receiving dihydroartemisinin-piperaquine alone (n=812).

Dashed lines: Boundaries of WHO recommended total dose for *P. falciparum*: 60-96 mg/kg if <25 kg, 48-81 mg/kg if ≥25 mg/kg.
