# Supplementary material for: The efficacy of dihydroartemisinin-piperaquine and artemether-lumefantrine with and without primaquine on Plasmodium vivax recurrence: A systematic review and individual patient data meta-analysis
Source: PLoS Med. 2019 Oct 4;16(10):e1002928. doi: 10.1371/journal.pmed.1002928 (PMC6777759; doi:10.1371/journal.pmed.1002928)
Supplement: S4 Fig — Risk of recurrence (derived from complement of Kaplan-Meier estimate) by study at days (A) 28, (B) 42, and (C) 63 in patients receiving dihydroartemisinin-piperaquine alone. (PDF) [file pmed.1002928.s007.pdf]

A. Day 28

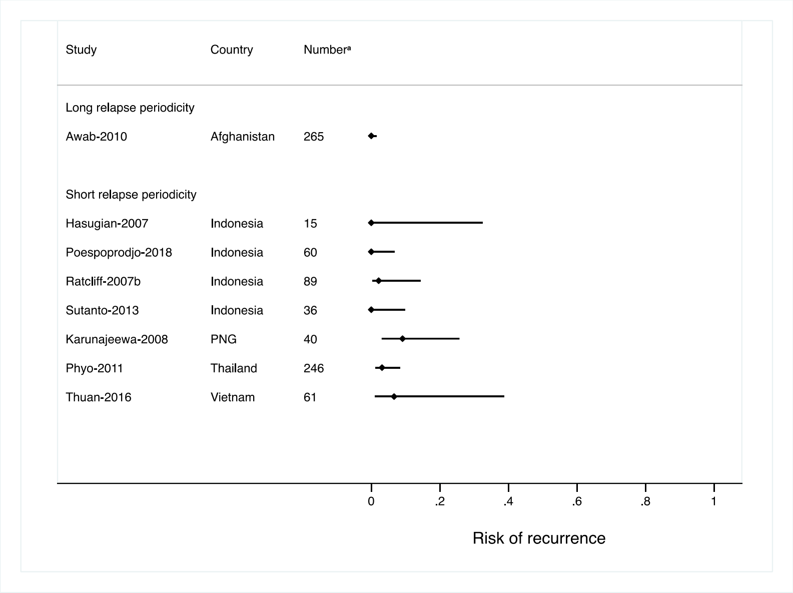

B. Day 42

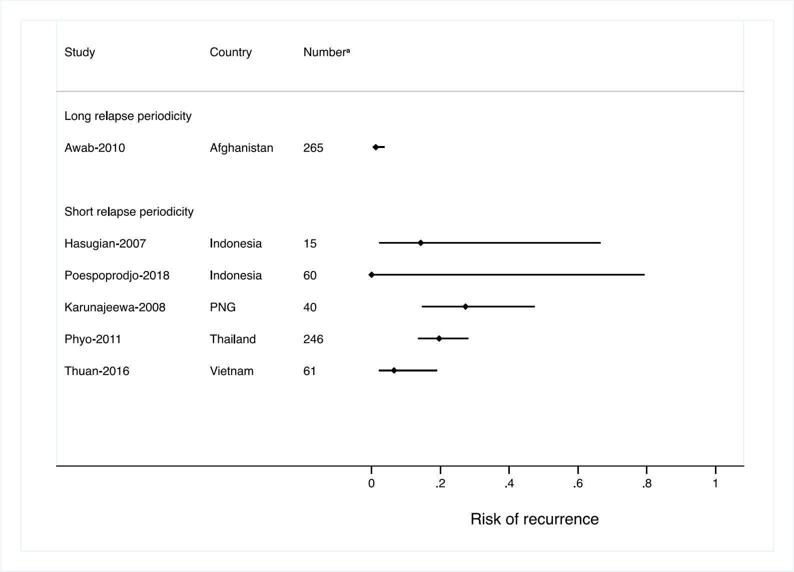

C. Day 63

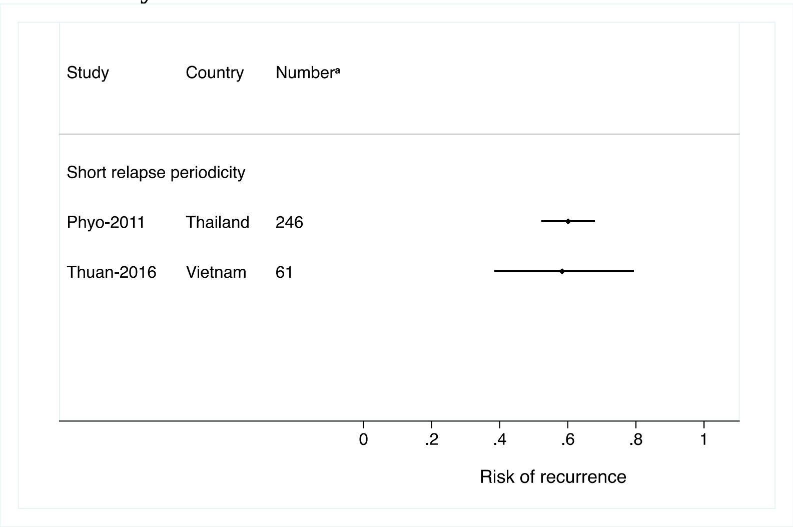

S4 Fig. Risk of recurrence (derived from complement of Kaplan-Meier estimate) by study at days (A) 28, (B) 42 and (C) 63 in patients receiving dihydroartemisinin-piperaquine alone

[a] Number refers to the total number of patients available for analysis per study for individuals treated with dihydroartemisinin-piperaquine alone

Where a Kaplan-Meier failure estimate for day X was not available due to no failures having occurred, confidence intervals were generated using Wilson's procedure for patients followed to day X. Sutanto *et al* did not have any failures, or any patients followed until day 42 or 63 [30].
