# Supplementary material for: The efficacy of dihydroartemisinin-piperaquine and artemether-lumefantrine with and without primaquine on Plasmodium vivax recurrence: A systematic review and individual patient data meta-analysis
Source: PLoS Med. 2019 Oct 4;16(10):e1002928. doi: 10.1371/journal.pmed.1002928 (PMC6777759; doi:10.1371/journal.pmed.1002928)
Supplement: S5 Fig — Histogram of drug dosing for (A) artemether in patients receiving artemether-lumefantrine alone (n = 384), (B) lumefantrine in patients receiving artemether-lumefantrine alone (n = 384), (C) artemether in patients receiving artemether-lumefantrine plus early primaquine (n = 208), (D) lumefantrine in patients receiving artemether-lumefantrine plus early primaquine (n = 208), and (E) primaquine in patients receiving artemether-lumefantrine plus early primaquine (n = 208). (PDF) [file pmed.1002928.s008.pdf]

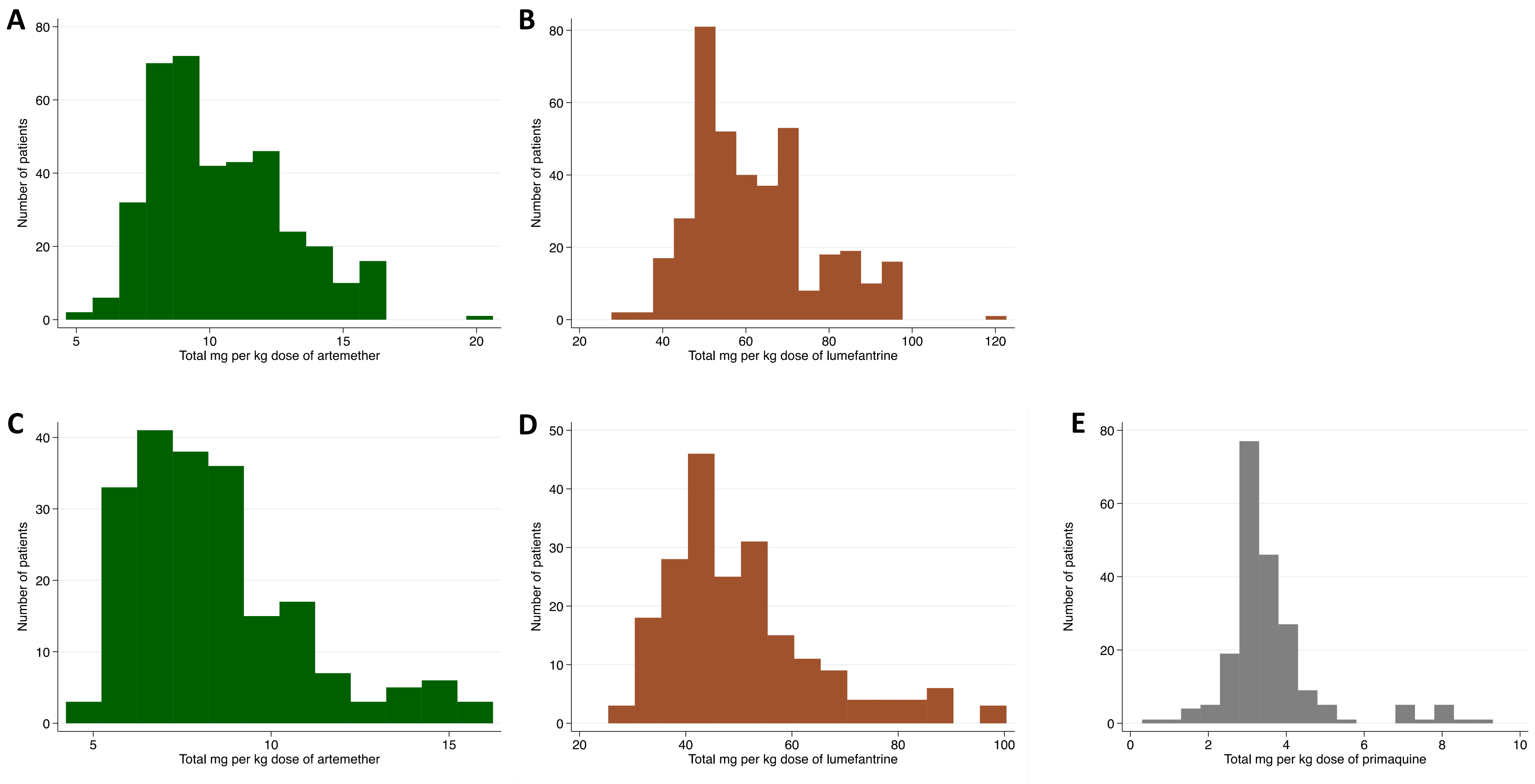

S5 Fig. Histogram of drug dosing for (A) artemether in patients receiving artemether-lumefantrine alone (n=384), (B) lumefantrine in patients receiving artemether-lumefantrine alone (n=384), (C) artemether in patients receiving artemether-lumefantrine plus early primaquine (n=208), (D) lumefantrine in patients receiving artemether-lumefantrine plus early primaquine (n=208) and (E) primaquine in patients receiving artemether-lumefantrine plus early primaquine (n=208).
