# Supplementary material for: The efficacy of dihydroartemisinin-piperaquine and artemether-lumefantrine with and without primaquine on Plasmodium vivax recurrence: A systematic review and individual patient data meta-analysis
Source: PLoS Med. 2019 Oct 4;16(10):e1002928. doi: 10.1371/journal.pmed.1002928 (PMC6777759; doi:10.1371/journal.pmed.1002928)
Supplement: S6 Fig — (PDF) [file pmed.1002928.s009.pdf]

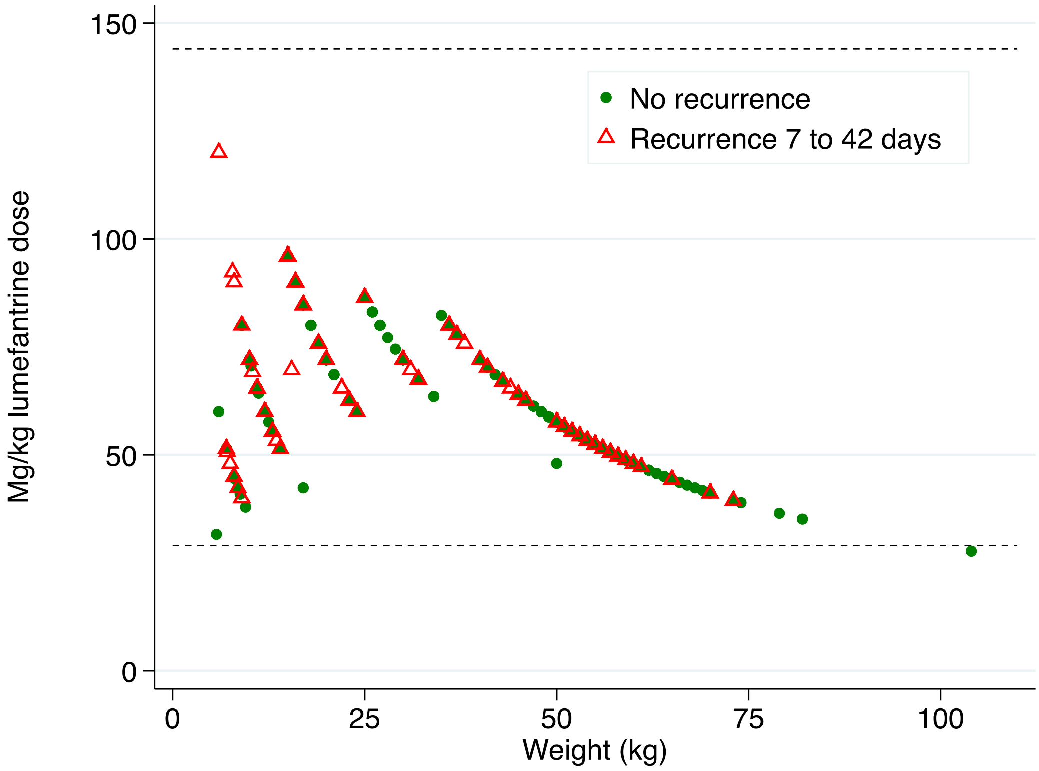

S6 Fig. Mg/kg total drug dosing of lumefantrine in patients receiving artemether-lumefantrine alone (n=384).

Dashed lines: Boundaries of WHO recommended total dose for *P. falciparum*: 29-144 mg/kg.
