# Supplementary material for: The efficacy of dihydroartemisinin-piperaquine and artemether-lumefantrine with and without primaquine on Plasmodium vivax recurrence: A systematic review and individual patient data meta-analysis
Source: PLoS Med. 2019 Oct 4;16(10):e1002928. doi: 10.1371/journal.pmed.1002928 (PMC6777759; doi:10.1371/journal.pmed.1002928)
Supplement: S7 Fig — (PDF) [file pmed.1002928.s010.pdf]

A. Day 28

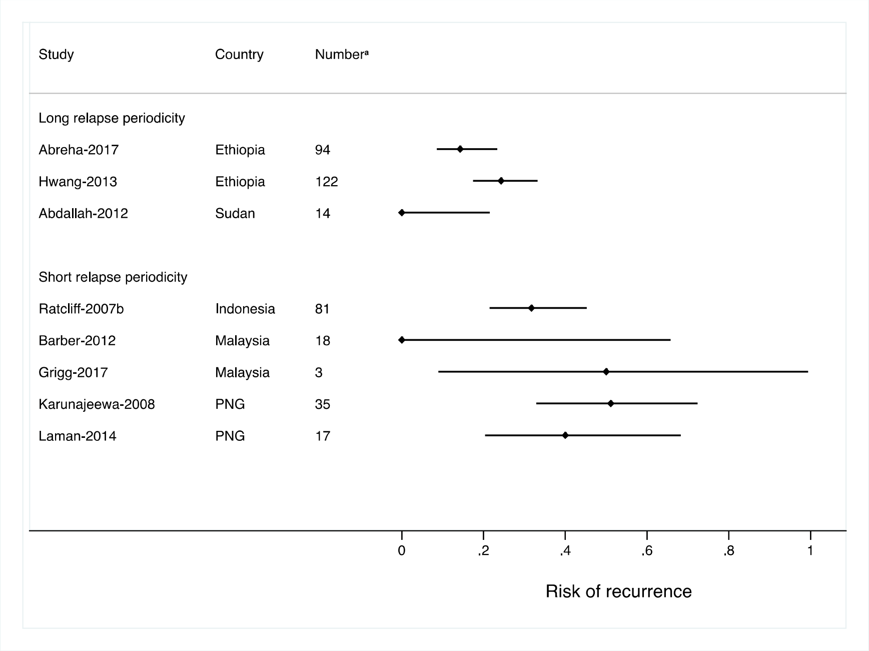

B. Day 42

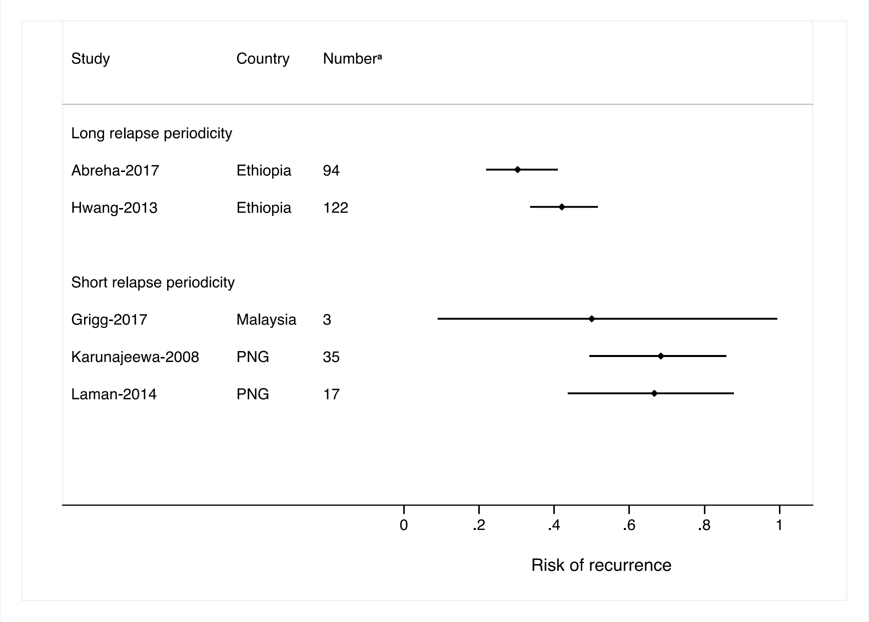

S7 Fig. Risk of recurrence by study at days 28 and 42 in patients receiving artemether-lumefantrine alone

[a] Number refers to the total number of patients available for analysis per study for individuals treated with artemether-lumefantrine alone

Where a Kaplan-Meier failure estimate for day 28 was not available due to no failures having occurred, confidence intervals were generated using Wilson's procedure for patients followed to day 28. Barber *et al* did not have any failures, or any patients followed until day 42 [28].
