# Supplementary material for: The efficacy of dihydroartemisinin-piperaquine and artemether-lumefantrine with and without primaquine on Plasmodium vivax recurrence: A systematic review and individual patient data meta-analysis
Source: PLoS Med. 2019 Oct 4;16(10):e1002928. doi: 10.1371/journal.pmed.1002928 (PMC6777759; doi:10.1371/journal.pmed.1002928)
Supplement: S1 Table — (PDF) [file pmed.1002928.s011.pdf]

S1 Table. Reasons for studies not being included in analysis

Reason	Number of studies	Studies[a]
No artemether-lumefantrine or dihydroartemisinin-piperaquine treatment arm	153	[5, 7, 56-206]
Intermittent primaquine dosing	1	[207]
Investigators unable to be contacted	1	[208]
Initial investigator response but no data provided	3	[209-211]
No response from investigators	4	[212-215]
Data available but no patients eligible	1	[55]

[a] References of studies not included are provided in S1 References.
