# Supplementary material for: The efficacy of dihydroartemisinin-piperaquine and artemether-lumefantrine with and without primaquine on Plasmodium vivax recurrence: A systematic review and individual patient data meta-analysis
Source: PLoS Med. 2019 Oct 4;16(10):e1002928. doi: 10.1371/journal.pmed.1002928 (PMC6777759; doi:10.1371/journal.pmed.1002928)
Supplement: S2 Table — (PDF) [file pmed.1002928.s012.pdf]

S2 Table. Studies included in analysis

Author-year	Country	Recruitment	Age included (years)			Follow up (days)	Treatment arms	Supervision	Drug origin	Patients enrolled[a]	Patients available[b]	Patients included[c]	AL Target (mg/kg)	DP Target (mg/kg)	Dose calculation method	PQ dosing	PQ timing	Published
			<5	5-15	>15													
Hasugian-2007 [25]	Indonesia	2005	Yes	Yes	Yes	42	AS+AQ±PQ, DP±PQ	Full ACT; Partial PQ	DP: Holley, China	152	152	45	-	6.75/54	Tablet count (PQ per protocol)	14 day × 0.3mg/kg	Day 2	Yes
Ratcliff-2007 [26]	Indonesia	2004-2005	Yes	Yes	Yes	42	AL, DP	Partial AL, Full DP	AL: Novartis, Switzerland<br>DP: Holleykin, China	288	288	170	Not specified	6.75/54	Tablet count	-	-	Yes
Karunajeewa-2008 [6]	Papua New Guinea	2005-2007	Yes	No	No	42	AL, DP, AS+SP, CQ+SP	All full except AL partial	AL: Novartis Pharma<br>DP: Holley-Cotec, China	195	195	75	10.4/60	7.5/60	Per protocol	-	-	Yes
Awab-2010 [38]	Afghanistan	2007-2009	Yes	Yes	Yes	56	CQ, DP	Full	DP: Holleypharm, China	536	536	265	-	6/48	Tablet count	-	-	Yes
Phyo-2011 [27]	Thailand	2007-2008	Yes	Yes	Yes	63	CQ, DP	Full	DP: Holley, China	492	498	246	-	7/55	Tablet count	14 day × 0.5mg/kg	End of Study	Yes
Abdallah-2012 [39]	Sudan	2011	Yes	Yes	Yes	28	AL+PQ	Partial AL, Partial PQ	AL: Novartis, Switzerland	43	38	38	Not specified	-	Per protocol	14 day × 15mg	Day 0	Yes
Barber-2013 [28]	Malaysia	2010-2011	No	Yes	Yes	42	AL, AL+PQ, varied	Not stated		43	86	31	Not specified	-	Tablet count	14 day × 0.5mg/kg	Day 0/1	Yes
Hwang-2013 [40]	Ethiopia	2009-2010	Yes	Yes	Yes	42	CQ, AL	Full CQ; Partial AL	AL: Novartis, Switzerland	242	242	122	Not specified	-	Per protocol	-	-	Yes
Pasaribu-2013 [29]	Indonesia	2010-2012	Yes	Yes	Yes	365	AS+AQ+PQ, DP+PQ	Full	DP: Pharbaco Central Pharmaceuticals, Vietnam<br>PQ: PT Phapros, Indonesia	331	331	164	-	6.75/54	Per protocol	14 day × 0.25mg/kg	Day 0	Yes
Sutanto-2013 [30]	Indonesia	2010-2011	No	No	Yes	365	DP+PQ, Qu+PQ, AS	Full	DP: Guilin Pharmaceutical<br>PQ: PT Phapros, Indonesia	116	115	36	-	Not specified	Tablet count	14 day × 0.5mg/kg	Day 28	Yes
Laman-2014 [31]	Papua New Guinea	2011-2013	Yes	No	No	42	AL, Art+N	Partial AL, Full Art+N	AL: Novartis, Switzerland	47	51	17	10.4/60	-	Tablet count	-	-	Yes
Lidia-2015 [32]	Indonesia	2013	No	No	Yes	42	CQ+PQ, DP+PQ	Full	DP: Kunming, China<br>PQ: PT Phapros, Indonesia	51	51	25	-	6-12/48-96	Tablet count	14 day × 0.25mg/kg	Day 0	Yes
Nelwan-2015 [33]	Indonesia	2013	No	No	Yes	365	DP+PQ, AS+PQ, AS+Pyr+PQ	Full	DP: Sigma-Tau, Italy<br>PQ: Sanofi-Aventis, Canada	180	180	57	-	Not specified	Tablet count	14 day × 0.5mg/kg	Day 0	Yes
Thuan-2016 [34]	Vietnam	2013-2014	Yes	Yes	Yes	63	CQ, DP	Full	DP: OPC Pharmaceutical, Vietnam	128	128	61	Not specified	-	Tablet count	14 day × 0.25mg/kg	End of Study	Yes
Abreha-2017 [41]	Ethiopia	2012-2016	Yes	Yes	Yes	365	AL, AL+PQ, CQ, CQ+PQ	Partial AL; Full CQ; Partial PQ	AL: Novartis, US<br>PQ: Sanofi-Aventis, US	398	398	181	Not specified	-	Tablet count	14 day × 0.25mg/kg	Day 2	Yes
Chu-2018 [35]	Thailand	2012-2014	Yes	Yes	Yes	365	DP+PQ, DP+PQ, CQ+PQ, CQ+PQ	Full	DP: Guilin Pharmaceutical, China<br>PQ: Government Pharmaceutical Org, Thailand	680	680	337	-	7/55	Per protocol	7 day × 1mg/kg, 14 day × 0.5mg/kg	Day 0	After literature search
Grigg-2018 [36]	Malaysia	2013-2016	No	Yes	Yes	42	AL, AL+PQ, varied	Partial AL; Partial PQ		-	56	4	Not specified	-	Tablet count	14 day × 0.5mg/kg	Day 0	After literature search
Daher-2018 [42]	Brazil	2012-2015	No	No	Yes	63	CQ+PQ, AL+PQ, AS+MQ+PQ	Partial AL, Partial PQ, CQ & AS+MQ full	AL: Novartis<br>PQ: Farmanguinhos-Fiocruz, Brazil	264	264	83	Not specified	-	Tablet count (PQ per protocol)	7-9 day × 0.5mg/kg	Day 0	Yes
Poespoprodjo-2018 [37]	Indonesia	2015-2016	Yes	Yes	Yes	42	DP+PQ	Full DP, PQ unsupervised		68	68	60	-	6.75/54	Tablet count (PQ per protocol)	14 day × 0.5mg/kg	Day 28	Yes

AL – artemether-lumefantrine; DP – dihydroartemisinin-piperaquine; PQ – primaquine; AS – artesunate; AQ – amodiaquine; SP – Sulfadoxine-pyrimethamine; CQ – chloroquine; Qu – quinine; Art – artemisinin; N – naphthoquine; Pyr – pyronaridine; MQ – mefloquine

[a] *P. vivax* patients enrolled in study as per manuscript; [b] Patients available for analysis from study; [c] Patients treated with AL or DP and included after exclusion criteria considered.
