# Supplementary material for: The efficacy of dihydroartemisinin-piperaquine and artemether-lumefantrine with and without primaquine on Plasmodium vivax recurrence: A systematic review and individual patient data meta-analysis
Source: PLoS Med. 2019 Oct 4;16(10):e1002928. doi: 10.1371/journal.pmed.1002928 (PMC6777759; doi:10.1371/journal.pmed.1002928)
Supplement: S3 Table — (PDF) [file pmed.1002928.s013.pdf]

S3 Table. Study sites included in analysis

Author-Year	Country	Study site	Latitude	Longitude	Year Start	Year End	MAP PvPR (%)[a]	Publication estimate of prevalence	Final prevalence classification	Region of relapse periodicity[a]	Final category of relapse periodicity[b]
Hasugian-2007 [25]	Indonesia	Timika	-4.50	136.85	2005	2005	4.7	Seasonal; API 403.34	High	12	High
Ratcliff-2007 [26]	Indonesia	Timika	-4.50	136.85	2005	2005	4.7	Seasonal; API 403.34	High	12	High
Karunajeewa-2008 [6]	Papua New Guinea	East Sepik	-4.46	143.22	2005	2007	7.8	Not stated	High	12	High
Karunajeewa-2008 [6]	Papua New Guinea	Madang	-5.23	145.79	2005	2007	9.4	Not stated	High	12	High
Awab-2010 [38]	Afghanistan	Jalalabad	34.43	70.46	2007	2009	1.4	Seasonal	Low	11	Low
Awab-2010 [38]	Afghanistan	Maimana	35.92	64.82	2007	2009	0.6	Seasonal	Low	11	Low
Awab-2010 [38]	Afghanistan	Taloqan	36.74	69.54	2007	2009	0.4	Seasonal	Low	11	Low
Phyo-2011 [27]	Thailand	Mae Sot	16.72	98.58	2007	2008	4.0	Low EIR <1; seasonal transmission	High	10	High
Abdallah-2012 [39]	Sudan	Kassala	15.41	36.41	2011	2011	0.4	Not stated	Low	7	Low
Barber-2013 [28]	Malaysia	Sabah	5.98	116.08	2010	2011	3.9	Not stated	Moderate	10	High
Hwang-2013 [40]	Ethiopia	Bishoftu	8.74	38.99	2009	2010	1.4	Seasonal; parasite prevalence rates of 0.3 and 0.9% in all ages and 0.6% in Oromia in 5-18 year olds	Low	7	Low
Hwang-2013 [40]	Ethiopia	Bulbula	7.72	38.65	2009	2010	2.2	Seasonal; parasite prevalence rates of 0.3 and 0.9% in all ages and 0.6% in Oromia in 5-18 year olds	Moderate	7	Low
Pasaribu-2013 [29]	Indonesia	Tanjung Leidong	2.33	100.05	2010	2012	0.3	400-500 per year amongst 32837 in 2010	Low	10	High
Sutanto-2013 [30]	Indonesia	Lumajang	-8.13	113.22	2010	2011	0.3	None - returned soldiers	Low	10	High
Laman-2014 [31]	Papua New Guinea	Madang	-5.23	145.79	2011	2013	9.4	Not stated	High	12	High
Lidia-2015 [32]	Indonesia	Kupang	-10.18	123.61	2013	2013	0.9	Not stated	Low	10	High
Nelwan-2015 [33]	Indonesia	Sragen	-7.42	111.02	2013	2014	0.3	None - returned soldiers	Low	10	High
Thuan-2016 [34]	Vietnam	Bu Gia Map	12.04	107.05	2013	2014	2.4	High	Moderate	10	High
Thuan-2016 [34]	Vietnam	Dak O	12.00	107.50	2013	2014	1.3	High	Moderate	10	High
Abreha-2017 [41]	Ethiopia	Bishoftu	8.73	39.01	2012	2016	1.4	Not available	Low	7	Low
Abreha-2017 [41]	Ethiopia	Batu	6.67	39.42	2012	2016	0.9	Not available	Low	7	Low
Chu-2018 [35]	Thailand	Mae Sot	16.72	98.58	2012	2014	4.0	Seasonal	High	10	High
Grigg-2018 [36]	Malaysia	Kudat	6.89	116.85	2013	2016	3.8	Not stated	Moderate	10	High
Daher-2018 [42]	Brazil	Rondonia	-3.15	-60.07	2012	2015	7.8	Not stated	High	3	Low
Daher-2018 [42]	Brazil	Manaus	-11.61	-63.56	2012	2015	7.4	Not stated	High	3	Low
Poespoprodjo-2018 [37]	Indonesia	Timika	-4.50	136.85	2005	2005	4.7	Unstable; prevalence 6.0% in 2013	High	12	High

API – annual parasite index; EIR – entomological inoculation rate

[a] *P. vivax* parasite rate estimated by Malaria Atlas Project (MAP) for 2010; [b] Short relapse periodicity ≤47 days
