# Supplementary material for: The efficacy of dihydroartemisinin-piperaquine and artemether-lumefantrine with and without primaquine on Plasmodium vivax recurrence: A systematic review and individual patient data meta-analysis
Source: PLoS Med. 2019 Oct 4;16(10):e1002928. doi: 10.1371/journal.pmed.1002928 (PMC6777759; doi:10.1371/journal.pmed.1002928)
Supplement: S4 Table — (PDF) [file pmed.1002928.s014.pdf]

S4 Table. Studies targeted for the analysis but not included

First Author	Treatment arms	Sites	Region	Country	Enrolled	Follow up (days)	Randomised	Recruitment period	Treatment arms	*Pv* patients enrolled	Pv treated with AL or DP	Female (%)	Age		Reason not included
													Mean (SD)	Median (range)	
Krudsood-2007 [212]	2	1	Asia-Pacific	Thailand	98	28	Yes	2004-2005	AL; CQ	98	47	25.5	25.1 (6.9)	- (15-65)	No response from investigators
Yohannes-2011 [213]	2	2	Africa	Ethiopia	159	28	No	2004-2005	AL; CQ	159	88	55.7	Not stated	17 (9.5-24)	No response from investigators
Tjitra-2012 [214]	2	2	Asia-Pacific	Indonesia	401	42	Yes	2007-2008	DP; Art+N	248	126	15.0	26.2 (9.2)	- (15-67)	No response from investigators
Eibach-2012 [208]	1	2	The Americas	Guyana	74	28	No	2009-2010	AL+PQ	74	74	9.5	Not stated	24 (5-57)	Investigator contact details unavailable
Senn-2013 [209]	1	1	Asia-Pacific	Papua New Guinea	926	42	No	2006-2010	AL	594	594	Not stated	Not stated	Not stated	Initial investigator response but no data provided
Leang-2013 [210]	1	4	Asia-Pacific	Cambodia	828	28	No	2008-2011	DP; CQ	390	173	28.3	22.9 (-)	- (3-60)	Initial investigator response but no data provided
Lon-2014 [211] [a]	1	2	Asia-Pacific	Cambodia	80	180	Yes	2010-2011	DP; DP	65	65	3.8	33.8 (-)	Not stated	Initial investigator response but no data provided
Shaikh-2017 [215]	1	1	Asia-Pacific	Pakistan	109	42	No	2012-2013	DP±PQ	109	109	39.0	Not stated	- (1-15)	No response from investigators

Pv – *P. vivax*; SD – standard deviation; AL – artemether-lumefantrine; CQ – chloroquine; DP – dihydroartemisinin-piperaquine; ART - artemisinin; N - naphthoquine; PQ – primaquine

[a] Sex and age estimates include 15 patients with *P. falciparum*.
