# Supplementary material for: The efficacy of dihydroartemisinin-piperaquine and artemether-lumefantrine with and without primaquine on Plasmodium vivax recurrence: A systematic review and individual patient data meta-analysis
Source: PLoS Med. 2019 Oct 4;16(10):e1002928. doi: 10.1371/journal.pmed.1002928 (PMC6777759; doi:10.1371/journal.pmed.1002928)
Supplement: S5 Table — (PDF) [file pmed.1002928.s015.pdf]

S5 Table. Comparison of baseline characteristics between included and targeted studies

Characteristic	Included studies (n=19)	Targeted studies[a] (n=8)
Region		
Asia-Pacific, studies (%)	15 (78.9%)	6 (75.0%)
Africa, studies (%)	3 (15.8%)	1 (12.5%)
The Americas, studies (%)	1 (5.3%)	1 (12.5%)
Year of enrolment[b]		
Pre-2011, studies (%)	6 (31.6%)	5 (62.5%)
2011-2017, studies (%)	13 (68.4%)	3 (37.5%)
Age, median (IQR)	18.0 (8.0, 30.0)	25.1 (22.9, 26.2)[c]
Female, % of patients	42.7%	28.3%[d]

[a] Age and female percentage of targeted studies frequency weighted according to number of patients treated with AL or DP; [b] Year of enrolment defined as the year study enrolment completed; [c] Mean age not available for four studies; [d] Percentage based on frequency weighted median of targeted studies, percentage not available from one study.
