# Supplementary material for: The efficacy of dihydroartemisinin-piperaquine and artemether-lumefantrine with and without primaquine on Plasmodium vivax recurrence: A systematic review and individual patient data meta-analysis
Source: PLoS Med. 2019 Oct 4;16(10):e1002928. doi: 10.1371/journal.pmed.1002928 (PMC6777759; doi:10.1371/journal.pmed.1002928)
Supplement: S6 Table — (PDF) [file pmed.1002928.s016.pdf]

S6 Table. Risk of bias assessment

Author-year	Randomisation	Allocation concealment	Blinding[a]	Bias related to participants included
Hasugian-2007 [26]	Yes - but only one treatment arm included	Block randomisation centrally; opaque sealed envelopes	No	Low risk[b]
Ratcliff-2007 [27]	Yes	Block randomisation centrally; opaque sealed envelopes	No	Low risk[b]
Karunajeewa-2008 [6]	Yes	Block randomisation by site	No	Low risk[b]
Awab-2010 [39]	Yes - but only one treatment arm included	Block randomisation centrally; opaque sealed envelopes	No	Low risk[b]
Phyo-2011 [28]	Yes - but only one treatment arm included	Block randomisation centrally; opaque sealed envelopes	No	Low risk[b]
Abdallah-2012 [40]	No	N/A	No	Moderate risk – data for 5/43 patients that were lost to follow up were not available
Barber-2013 [29]	No	N/A	No	Low risk[b]
Hwang-2013 [41]	Yes - but only one treatment arm included	Randomisation centrally; sealed envelopes	No	Low risk[b]
Pasaribu-2013 [30]	Yes - but only one treatment arm included	Block randomisation centrally; opaque sealed envelopes	No	Low risk[b]
Sutanto-2013 [31]	Yes	Block randomisation centrally; opaque sealed envelopes	No	Low risk – data for 1/116 patients that withdrew consent prior to treatment was unavailable
Laman-2014 [32]	Yes - but only one treatment arm included	Block randomisation centrally; opaque sealed envelopes	No	Low risk[b]
Lidia-2015 [33]	No	N/A	No	Low risk[b]
Nelwan-2015 [34]	Yes - but only one treatment arm included	Block randomisation centrally; opaque sealed envelopes	No	Low risk[b]
Thuan-2016 [35]	Yes - but only one treatment arm included	Block randomisation centrally; opaque sealed envelopes	No	Low risk[b]
Abreha-2017 [42]	Yes	Block randomisation centrally; opaque sealed envelopes	No	Low risk[b]
Chu-2018 [36]	Yes	Block randomisation centrally	No	Low risk[b]
Grigg-2018 [37]	No	N/A	No	Low risk[b]
Daher-2018 [4]	Yes	Block randomisation centrally; opaque sealed envelopes	No	Low risk[b]
Poespoprodjo-2018 [38]	No	N/A	No	Low risk[b]

[a]Blinding refers to the blinding of the patient and clinician to treatment.

[b]All individual patient data available and curated and standardised with re-analysis according to the WWARN Data Management and Statistical Analysis plan [17].

Bias related to assessment of outcomes was considered to be low risk as studies were efficacy studies with recurrent parasitaemia as their primary outcomes, consistent with the outcome of the current meta-analysis; Bias related to time to event outcome data has been minimised in the current analysis by extending analysis beyond day 42 where possible.
