# Supplementary material for: The efficacy of dihydroartemisinin-piperaquine and artemether-lumefantrine with and without primaquine on Plasmodium vivax recurrence: A systematic review and individual patient data meta-analysis
Source: PLoS Med. 2019 Oct 4;16(10):e1002928. doi: 10.1371/journal.pmed.1002928 (PMC6777759; doi:10.1371/journal.pmed.1002928)
Supplement: S7 Table — (PDF) [file pmed.1002928.s017.pdf]

S7 Table. Sensitivity analysis investigating the effect of piperaquine dose on the rate of *P. vivax* recurrence between days 7 and 42 for patients that received dihydroartemisinin-piperaquine alone

Variable	Range of AHR	Coefficient of Variation (%)[a]
Piperaquine dose, per every 5 mg/kg increase	0.60-0.75	6.08
Age, years		
≥15	1	-
<5	1.53-2.32	11.87
5 to <15	0.49-2.03	49.76
Gender		
Male	1	-
Female	0.58-0.67	3.83
Parasitaemia, parasites per μL every ten-fold increase	1.11-1.39	5.81
Haemoglobin, g/dL	0.80-0.84	1.38
Relapse periodicity		
Long	1	-
Short[b]	$15.82\text{-}2.1\times10^{17}$	346.41

AHR – Adjusted hazard ratio

Sensitivity analysis was generated by removing each of the 12 study sites one at a time.

[a] The coefficient of variation calculated as standard deviation divided by the mean of the estimates; [b] One study site (Maimana, Afghanistan, Awab-2010 [38]) with 47 patients included two of the three recurrences from low relapse periodicity regions leading to an increased adjusted hazard ratio for short relapse periodicity regions when this site was excluded. The AHR range excluding this site was 15.82-34.98 and the coefficient of variation was 21.87%.
