# Supplementary material for: The efficacy of dihydroartemisinin-piperaquine and artemether-lumefantrine with and without primaquine on Plasmodium vivax recurrence: A systematic review and individual patient data meta-analysis
Source: PLoS Med. 2019 Oct 4;16(10):e1002928. doi: 10.1371/journal.pmed.1002928 (PMC6777759; doi:10.1371/journal.pmed.1002928)
Supplement: S8 Table — (PDF) [file pmed.1002928.s018.pdf]

S8 Table. Sensitivity analysis investigating the effect of schizontocidal treatment on the rate of *P. vivax* recurrence between days 7 and 42 for patients that received dihydroartemisinin-piperaquine or artemether-lumefantrine alone

**Variable**	**Range of AHR**	**Coefficient of Variation (%)**[a]
Treatment		
Dihydroartemisinin-piperaquine	1	-
Artemether-lumefantrine	9.88-19.54	17.78
Age, per every 5 year increase	0.91-0.96	1.05
Gender		
Male	1	-
Female	0.62-0.85	5.76
Parasitaemia, parasites per µL every ten-fold increase	1.36-1.56	3.33
Haemoglobin, g/dL	0.83-0.88	1.16
Relapse periodicity		
Long	1	-
Short	1.69-4.13	17.97

AHR – Adjusted hazard ratio; Sensitivity analysis was generated by removing each of the 20 study sites one at a time.

[a] The coefficient of variation calculated as standard deviation divided by the mean of the estimates.
