# Supplementary material for: The efficacy of dihydroartemisinin-piperaquine and artemether-lumefantrine with and without primaquine on Plasmodium vivax recurrence: A systematic review and individual patient data meta-analysis
Source: PLoS Med. 2019 Oct 4;16(10):e1002928. doi: 10.1371/journal.pmed.1002928 (PMC6777759; doi:10.1371/journal.pmed.1002928)
Supplement: S9 Table — (PDF) [file pmed.1002928.s019.pdf]

S9 Table. Multivariable models for effect of primaquine use on the rate of *P. vivax* recurrence between days 7 and 42 in patients receiving dihydroartemisinin-piperaquine or artemether lumefantrine

	**Dihydroartemisinin-piperaquine**			**Artemether-lumefantrine**		
	**Total N (n)[a]**	**Adjusted HR (95% CI)**	**p value**	**Total N (n)[a]**	**Adjusted HR (95% CI)**	**p value**
Primaquine						
No	764 (41)	Reference	-	333 (119)	Reference	-
Yes	612 (3)	0.23 (0.04, 1.28)	0.0933	184 (9)	0.20 (0.10, 0.41)	<0.0001
Piperaquine dose, per every 5 mg/kg increase	1376 (44)	0.56 (0.43, 0.73)	<0.0001	-	-	-
Lumefantrine dose, per every 5 mg/kg increase	-	-	-	517 (128)	1.04 (0.97, 1.12)	0.2347
Age, per every 5 year increase	1376 (44)	1.00 (0.89, 1.12)	0.9432	517 (128)	0.91 (0.83, 1.00)	0.0615
Gender						
Male	760 (32)	Reference	-	329 (76)	Reference	-
Female	616 (12)	0.69 (0.35, 1.36)	0.2794	188 (52)	0.83 (0.57, 1.20)	0.3209
Parasitaemia, parasites per µL every ten-fold increase	1376 (44)	1.27 (0.80, 2.02)	0.3193	517 (128)	1.41 (1.02, 1.97)	0.0394
Baseline haemoglobin, per 1 g/dL increase	1376 (44)	0.78 (0.66, 0.92)	0.0040	517 (128)	0.90 (0.82, 0.99)	0.0239
Relapse periodicity						
Long	264 (2)	Reference	-	386 (84)	Reference	-
Short	1112 (42)	28.48 (3.34, 242.45)	0.0022	131 (44)	1.35 (0.76, 2.41)	0.3022

HR – hazard ratio. CI = Confidence Interval.

[a] Number of patients (number with recurrence by day 42); [b] AHR unable to be estimated.

Dihydroartemisinin-piperaquine – theta (variance of frailty parameter for clustering of study sites) = 1.19; Artemether-lumefantrine – theta = 0.03.

The assumption of proportional hazards held for both models (global test 0.1345 for dihydroartemisinin-piperaquine and 0.3651 for artemether-lumefantrine).

To examine the robustness of the parameter estimates, a sensitivity analysis was carried out by removing one study site at a time which showed that the overall coefficient of variation of parameter estimates in the multivariable models was relatively minimal (S12 Table).
