# Supplementary material for: The efficacy of dihydroartemisinin-piperaquine and artemether-lumefantrine with and without primaquine on Plasmodium vivax recurrence: A systematic review and individual patient data meta-analysis
Source: PLoS Med. 2019 Oct 4;16(10):e1002928. doi: 10.1371/journal.pmed.1002928 (PMC6777759; doi:10.1371/journal.pmed.1002928)
Supplement: S10 Table — (PDF) [file pmed.1002928.s020.pdf]

S10 Table. Multivariable models for effect of primaquine dose on the rate of *P. vivax* recurrence between days 7 and 42 in patients receiving dihydroartemisinin-piperaquine or artemether lumefantrine

	**Dihydroartemisinin-piperaquine**			**Artemether-lumefantrine**		
	**Total N (n)[a]**	**Adjusted HR (95% CI)**	**p value**	**Total N (n)[a]**	**Adjusted HR (95% CI)**	**p value**
Primaquine						
No	764 (41)	Reference	-	333 (119)	Reference	-
Low dose	173 (2)	0.28 (0.04, 1.99)	0.2031	167 (9)	0.21 (0.10, 0.43)	<0.0001
High dose	439 (1)	0.15 (0.01, 1.96)	0.1494	17 (0)[b]	-	-
Piperaquine dose, per every 5 mg/kg increase	1376 (44)	0.56 (0.43, 0.73)	<0.0001	-	-	-
Lumefantrine dose, per every 5 mg/kg increase	-	-	-	517 (128)	1.04 (0.97, 1.12)	0.2280
Age, per every 5 year increase	1376 (44)	1.00 (0.88, 1.12)	0.9346	517 (128)	0.91 (0.83, 1.00)	0.0628
Gender						
Male	760 (32)	Reference	-	329 (76)	Reference	-
Female	616 (12)	0.69 (0.35, 1.37)	0.2933	188 (52)	0.83 (0.57, 1.19)	0.3099
Parasitaemia, parasites per µL every ten-fold increase	1376 (44)	1.26 (0.79, 2.01)	0.3239	517 (128)	1.42 (1.02, 1.97)	0.0385
Baseline haemoglobin, per 1 g/dL increase	1376 (44)	0.78 (0.66, 0.92)	0.0040	517 (128)	0.90 (0.82, 0.99)	0.0242
Relapse periodicity						
Long	264 (2)	Reference	-	386 (84)	Reference	-
Short	1112 (42)	28.94 (3.48, 240.90)	0.0019	131 (44)	1.37 (0.77, 2.43)	0.2841

HR – hazard ratio. CI = Confidence Interval

[a] Number of patients (number with recurrence by day 42); [b] AHR unable to be estimated.

Dihydroartemisinin-piperaquine – theta (variance of frailty parameter for clustering of study sites) = 1.13; Artemether-lumefantrine – theta = 0.03.

The assumption of proportional hazards held for both models by visual inspection.
