# Supplementary material for: The efficacy of dihydroartemisinin-piperaquine and artemether-lumefantrine with and without primaquine on Plasmodium vivax recurrence: A systematic review and individual patient data meta-analysis
Source: PLoS Med. 2019 Oct 4;16(10):e1002928. doi: 10.1371/journal.pmed.1002928 (PMC6777759; doi:10.1371/journal.pmed.1002928)
Supplement: S11 Table — (PDF) [file pmed.1002928.s021.pdf]

S11 Table. Multivariable models for effect of primaquine on the rate of *P. vivax* recurrence between days 7 and 63 in patients receiving dihydroartemisinin-piperaquine

	Total N (n)[a]	Adjusted HR (95% CI)	p value
Primaquine			
No	341 (133)	Reference	-
Yes	221 (2)	0.06 (0.01, 0.63)	0.0196
Piperaquine dose, per every 5 mg/kg increase	562 (135)	0.76 (0.65, 0.88)	0.0002
Age, per every 5 year increase	562 (135)	0.99 (0.98, 1.01)	0.2781
Gender			
Male	309 (98)	Reference	-
Female	253 (37)	0.67 (0.45, 1.01)	0.0567
Parasitaemia, parasites per µL every ten-fold increase	562 (135)	1.42 (1.07, 1.87)	0.0151
Baseline haemoglobin, per 1 g/dL increase	562 (135)	0.95 (0.86, 1.05)	0.3605
Relapse periodicity			
Long	0 (0)	Reference	-
Short	562 (135)	-	-

HR – hazard ratio. CI = Confidence Interval.

[a] Number of patients (number with recurrence by day 63). Only includes studies where patients followed for 63 days or longer. No patients from regions of long relapse periodicity were available for inclusion.

Theta (variance of frailty parameter for clustering of study sites) = 1.17.

The assumption of proportional hazards held by visual inspection.
