# Supplementary material for: The efficacy of dihydroartemisinin-piperaquine and artemether-lumefantrine with and without primaquine on Plasmodium vivax recurrence: A systematic review and individual patient data meta-analysis
Source: PLoS Med. 2019 Oct 4;16(10):e1002928. doi: 10.1371/journal.pmed.1002928 (PMC6777759; doi:10.1371/journal.pmed.1002928)
Supplement: S12 Table — (PDF) [file pmed.1002928.s022.pdf]

S12 Table. Sensitivity analysis investigating the effect of primaquine use on the rate of *P. vivax* recurrence between days 7 and 42

Variable	Dihydroartemisinin-piperaquine		Artemether-lumefantrine	
	Range of AHR	Coefficient of Variation (%)[a]	Range of AHR	Coefficient of Variation (%)[a]
Primaquine				
No	1	-	1	-
Yes	0.00-0.37	32.97	0.14-0.31	13.46
Piperaquine dose, per every 5 mg/kg increase	0.52-0.66	5.09	-	-
Lumefantrine dose, per every 5 mg/kg increase	-	-	0.96-1.05	1.59
Age, per every 5 year increase	0.99-1.01	0.40	0.89-0.93	1.01
Gender				
Male	1	-	1	-
Female	0.62-0.86	7.16	0.72-0.96	4.91
Parasitaemia, parasites per µL every ten-fold increase	1.19-1.51	5.43	1.32-1.74	5.18
Haemoglobin, g/dL	0.76-0.80	1.01	0.88-0.94	1.21
Relapse periodicity				
Long	1	-	1	-
Short	17.64-5.6x10^{16} [b]	400.00	0.89-2.25	15.17

AHR – Adjusted hazard ratio; Sensitivity analyses were generated by removing each of the 16 study sites with dihydroartemisinin-piperaquine one at a time and each of the 26 study sites with artemether-lumefantrine one at a time.

[a] The coefficient of variation calculated as standard deviation divided by the mean of the estimates; [b] The AHR for short relapse periodicity ranged between 17.64 and 34.52 with removal of all studies except for Maimana, Afghanistan (Awab-2010) where the AHR following removal was 5.6x10^{16} due to two of three recurrences from long relapse periodicity regions occurring in this study.
