# Supplementary material for: The efficacy of dihydroartemisinin-piperaquine and artemether-lumefantrine with and without primaquine on Plasmodium vivax recurrence: A systematic review and individual patient data meta-analysis
Source: PLoS Med. 2019 Oct 4;16(10):e1002928. doi: 10.1371/journal.pmed.1002928 (PMC6777759; doi:10.1371/journal.pmed.1002928)
Supplement: S13 Table — (PDF) [file pmed.1002928.s023.pdf]

S13 Table. Multivariable models for effect of day of recurrence on the haemoglobin at recurrence in patients treated with artemether-lumefantrine with or without primaquine

	Any recurrence			Symptomatic recurrence		
	**Total[a]**	**Coefficient, g/dL (95% CI)**	**p value**	**Total[b]**	**Coefficient, g/dL (95% CI)**	**p value**
Day of recurrence, per 5 day increase	109	0.07 (-0.06, 0.20)	0.3130	70	0.13 (0.01, 0.26)	0.0407
Baseline haemoglobin, per 1 g/dL increase	109	0.35 (0.24, 0.46)	<0.0001	70	0.50 (0.38, 0.63)	<0.0001
Age, per every 5 year increase	109	0.08 (-0.02, 0.19)	0.1123	70	0.00 (-0.02, 0.03)	0.6639
Gender						
Male	61	Reference	-	41	Reference	-
Female	48	0.02 (-0.44, 0.48)	0.9379	29	0.00 (-0.47, 0.48)	0.9981
Baseline parasitaemia, parasites per µL every ten-fold increase	109	-0.11 (-0.58, 0.35)	0.6327	70	-0.33 (-0.79, 0.13)	0.1557
Parasitaemia at recurrence, parasites per µL every ten-fold increase	109	0.05 (-0.23, 0.34)	0.7069	70	0.14 (-0.18, 0.46)	0.3779
Relapse periodicity						
Long	86	Reference	-	59	Reference	-
Short	23	-0.91 (-1.53, -0.28)	0.0043	11	-0.28 (-0.99, 0.43)	0.4396
Primaquine						
No	101	Reference	-	65	Reference	-
Yes	8	-0.34 (-1.20, 0.51)	0.4274	5	-0.67 (-1.59, 0.24)	0.1500

CI = Confidence Interval

[a] Number of patients with any recurrence between day 7 and day 63.

[b] Number of patients with a symptomatic recurrence between day 7 and day 63.
