# Supplementary material for: The efficacy of dihydroartemisinin-piperaquine and artemether-lumefantrine with and without primaquine on Plasmodium vivax recurrence: A systematic review and individual patient data meta-analysis
Source: PLoS Med. 2019 Oct 4;16(10):e1002928. doi: 10.1371/journal.pmed.1002928 (PMC6777759; doi:10.1371/journal.pmed.1002928)
Supplement: S1 References — (PDF) [file pmed.1002928.s024.pdf]

S1 References. References for studies identified in the systematic review but not included in the analysis

5. Sumawinata IW, Bernadeta, Leksana B, Sutamihardja A, Purnomo, Subianto B, et al. Very high risk of therapeutic failure with chloroquine for uncomplicated *Plasmodium falciparum* and *P. vivax* malaria in Indonesian Papua. Am J Trop Med Hyg. 2003;68(4):416-20. Epub 2003/07/24. PubMed PMID: 12875290.

7. Ratcliff A, Siswantoro H, Kenangalem E, Wuwung M, Brockman A, Edstein MD, et al. Therapeutic response of multidrug-resistant *Plasmodium falciparum* and *P. vivax* to chloroquine and sulfadoxine-pyrimethamine in southern Papua, Indonesia. Trans R Soc Trop Med Hyg. 2007;101(4):351-9. Epub 2006/10/10. doi: 10.1016/j.trstmh.2006.06.008. PubMed PMID: 17028048; PubMed Central PMCID: PMCPMC2080856.

56. Alecrim MG, Carvalho LM, Fernandes MC, de Andrade SD, Loureiro AC, Arcanjo AR, et al. [Malaria treatment with artesunate (retocaps) in children of the Brazilian Amazon]. Rev Soc Bras Med Trop. 2000;33(2):163-8. Epub 2000/07/06. PubMed PMID: 10881128.

57. Bergonzoli G, Rivers Cuadra JC. [Therapeutic efficacy of different antimalarial regimens in the Costa Rica-Nicaragua border region]. Rev Panam Salud Publica. 2000;7(6):366-70. Epub 2000/08/19. PubMed PMID: 10949896.

58. Pukrittayakamee S, Chantra A, Simpson JA, Vanijanonta S, Clemens R, Looareesuwan S, et al. Therapeutic responses to different antimalarial drugs in vivax malaria. Antimicrob Agents Chemother. 2000;44(6):1680-5. Epub 2000/05/19. PubMed PMID: 10817728; PubMed Central PMCID: PMCPMC89932.

59. Singh RK. Emergence of chloroquine-resistant vivax malaria in south Bihar (India). Trans R Soc Trop Med Hyg. 2000;94(3):327. Epub 2000/09/07. PubMed PMID: 10975013.

60. Taylor WR, Doan HN, Nguyen DT, Tran TU, Fryauff DJ, Gomez-Saladin E, et al. Assessing drug sensitivity of *Plasmodium vivax* to halofantrine or choroquine in southern, central Vietnam using an extended 28-day *in vivo* test and polymerase chain reaction genotyping. Am J Trop Med Hyg. 2000;62(6):693-7. Epub 2001/04/17. PubMed PMID: 11304056.

61. Villalobos-Salcedo JM, Tada MS, Kimura E, Menezes MJ, Pereira da Silva LH. *In-vivo* sensitivity of *Plasmodium vivax* isolates from Rondnia (western Amazon region, Brazil) to regimens including chloroquine and primaquine. Ann Trop Med Parasitol. 2000;94(8):749-58. Epub 2001/02/24. PubMed PMID: 11214093.

62. Abdon NP, Pinto AY, das Silva Rdo S, de Souza JM. [Assessment of the response to reduced treatment schemes for vivax malaria]. Rev Soc Bras Med Trop. 2001;34(4):343-8. Epub 2001/09/20. PubMed PMID: 11562727.

63. Adak T, Valecha N, Sharma VP. *Plasmodium vivax* polymorphism in a clinical drug trial. Clin Diagn Lab Immunol. 2001;8(5):891-4. Epub 2001/08/31. doi: 10.1128/CDLI.8.5.891-894.2001. PubMed PMID: 11527798; PubMed Central PMCID: PMCPMC96166.

64. Buchachart K, Krudsood S, Singhasivanon P, Treeprasertsuk S, Phophak N, Srivilairit S, et al. Effect of primaquine standard dose (15 mg/day for 14 days) in the treatment of vivax malaria patients in Thailand. Southeast Asian J Trop Med Public Health. 2001;32(4):720-6. Epub 2002/06/04. PubMed PMID: 12041544.

65. Dua VK, Sharma VP. *Plasmodium vivax* relapses after 5 days of primaquine treatment, in some industrial complexes of India. Ann Trop Med Parasitol. 2001;95(7):655-9. Epub 2002/01/11. doi: 10.1080/00034980120103225. PubMed PMID: 11784418.

66. Duarte EC, Pang LW, Ribeiro LC, Fontes CJ. Association of subtherapeutic dosages of a standard drug regimen with failures in preventing relapses of vivax malaria. Am J Trop Med Hyg. 2001;65(5):471-6. PubMed PMID: 11716100.

67. Pukrittayakamee S, Clemens R, Chantra A, Nontprasert A, Luknam T, Looareesuwan S, et al. Therapeutic responses to antibacterial drugs in vivax malaria. Trans R Soc Trop Med Hyg. 2001;95(5):524-8. Epub 2001/11/15. PubMed PMID: 11706666.

68. Soto J, Toledo J, Gutierrez P, Luzz M, Llinas N, Cedeno N, et al. *Plasmodium vivax* clinically resistant to chloroquine in Colombia. Am J Trop Med Hyg. 2001;65(2):90-3. Epub 2001/08/18. PubMed PMID: 11508397.

69. Taylor WR, Widjaja H, Richie TL, Basri H, Ohrt C, Tjitra, et al. Chloroquine/doxycycline combination versus chloroquine alone, and doxycycline alone for the treatment of *Plasmodium falciparum* and *Plasmodium vivax* malaria in northeastern Irian Jaya, Indonesia. Am J Trop Med Hyg. 2001;64(5-6):223-8. Epub 2001/07/21. PubMed PMID: 11463107.

70. Baird JK, Tiwari T, Martin GJ, Tamminga CL, Prout TM, Tjaden J, et al. Chloroquine for the treatment of uncomplicated malaria in Guyana. Ann Trop Med Parasitol. 2002;96(4):339-48. Epub 2002/08/13. doi: 10.1179/000349802125001023. PubMed PMID: 12171615.

71. Castillo CM, Osorio LE, Palma GI. Assessment of therapeutic response of *Plasmodium vivax* and *Plasmodium falciparum* to chloroquine in a Malaria transmission free area in Colombia. Mem Inst Oswaldo Cruz. 2002;97(4):559-62. Epub 2002/07/16. PubMed PMID: 12118291.

72. Congpuong K, Na-Bangchang K, Thimasarn K, Tasanor U, Wernsdorfer WH. Sensitivity of *Plasmodium vivax* to chloroquine in Sa Kaeo Province, Thailand. Acta Trop. 2002;83(2):117-21. Epub 2002/06/29. PubMed PMID: 12088852.

73. Fryauff DJ, Leksana B, Masbar S, Wiady I, Sismadi P, Susanti AI, et al. The drug sensitivity and transmission dynamics of human malaria on Nias Island, North Sumatra, Indonesia. Ann Trop Med Parasitol. 2002;96(5):447-62. Epub 2002/08/27. doi: 10.1179/000349802125001249. PubMed PMID: 12194705.

74. Hamedi Y, Nateghpour M, Tan-ariya P, Tiensuwan M, Silachamroon U, Looareesuwan S. *Plasmodium vivax* malaria in Southeast Iran in 1999-2001: establishing the response to chloroquine *in vitro* and *in vivo*. Southeast Asian J Trop Med Public Health. 2002;33(3):512-8. Epub 2003/04/16. PubMed PMID: 12693585.

75. Maguire JD, Lacy MD, Sururi, Sismadi P, Krisin, Wiady I, et al. Chloroquine or sulfadoxine-pyrimethamine for the treatment of uncomplicated, *Plasmodium falciparum* malaria during an epidemic in Central Java, Indonesia. Ann Trop Med Parasitol. 2002;96(7):655-68. Epub 2003/01/23. doi: 10.1179/000349802125002310. PubMed PMID: 12537627.

76. Lacy MD, Maguire JD, Barcus MJ, Ling J, Bangs MJ, Gramzinski R, et al. Atovaquone/proguanil therapy for *Plasmodium falciparum* and *Plasmodium vivax* malaria in Indonesians who lack clinical immunity. Clin Infect Dis. 2002;35(9):e92-5. Epub 2002/10/18. doi: 10.1086/343750. PubMed PMID: 12384852.

77. McGready R, Thwai KL, Cho T, Samuel, Looareesuwan S, White NJ, et al. The effects of quinine and chloroquine antimalarial treatments in the first trimester of pregnancy. Trans R Soc Trop Med Hyg. 2002;96(2):180-4. Epub 2002/06/12. PubMed PMID: 12055810.

78. Mohapatra MK, Padhiary KN, Mishra DP, Sethy G. Atypical manifestations of *Plasmodium vivax* malaria. Indian J Malariol. 2002;39(1-2):18-25. Epub 2003/12/23. PubMed PMID: 14686106.

79. Phan GT, de Vries PJ, Tran BQ, Le HQ, Nguyen NV, Nguyen TV, et al. Artemisinin or chloroquine for blood stage *Plasmodium vivax* malaria in Vietnam. Trop Med Int Health. 2002;7(10):858-64. Epub 2002/10/03. PubMed PMID: 12358621.

80. Tjitra E, Baker J, Suprianto S, Cheng Q, Anstey NM. Therapeutic efficacies of artesunate-sulfadoxine-pyrimethamine and chloroquine-sulfadoxine-pyrimethamine in vivax malaria pilot studies: relationship to *Plasmodium vivax dhfr* mutations. Antimicrob Agents Chemother. 2002;46(12):3947-53. Epub 2002/11/19. PubMed PMID: 12435700; PubMed Central PMCID: PMCPMC132782.

81. Blair-Trujillo S, Castano AT, Restrepo ME, Sanchez GA, Carmona-Fonseca J. [Adequate clinical and parasitological *Plasmodium vivax* response to chloroquine in Colombia (Turbo, Antioquia), 2001]. Infectio. 2002;6(1):21-6.

82. Yadav RS, Ghosh SK. Radical curative efficacy of five-day regimen of primaquine for treatment of *Plasmodium vivax* malaria in India. J Parasitol. 2002;88(5):1042-4. Epub 2002/11/19. doi: 10.1645/0022-3395(2002)088[1042:RCEOFD]2.0.CO;2. PubMed PMID: 12435158.

83. da Silva Rdo S, Pinto AY, Calvosa VS, de Souza JM. [Short course schemes for vivax malaria treatment]. Rev Soc Bras Med Trop. 2003;36(2):235-9. Epub 2003/06/14. PubMed PMID: 12806460.

84. Fernandopulle BM, Weeraratne CL, Weerasuriya K, Karunaweera ND. Efficacy of a five-day course of primaquine in preventing relapses in *Plasmodium vivax* malaria--a pilot study. Ceylon Med J. 2003;48(1):32. Epub 2003/06/11. PubMed PMID: 12795021.

85. Machado RL, de Figuereido Filho AF, Calvosa VS, Figueredo MC, Nascimento JM, Povoa MM. Correlation between *Plasmodium vivax* variants in Belem, Para State, Brazil and symptoms and clearance of parasitaemia. Braz J Infect Dis. 2003;7(3):175-7. Epub 2003/09/23. PubMed PMID: 14499040.

86. Nandy A, Addy M, Maji AK, Bandyopadhyay AK. Monitoring the chloroquine sensitivity of *Plasmodium vivax* from Calcutta and Orissa, India. Ann Trop Med Parasitol. 2003;97(3):215-20. Epub 2003/06/14. doi: 10.1179/000349803235001868. PubMed PMID: 12803853.

87. Pinto AY, Azevedo CH, da Silva JB, de Souza JM. Assessment of chloroquine single dose treatment of malaria due to *Plasmodium vivax* in Brazilian Amazon. Rev Inst Med Trop Sao Paulo. 2003;45(6):327-31. Epub 2004/02/06. PubMed PMID: 14762633.

88. Rajgor DD, Gogtay NJ, Kadam VS, Kamtekar KD, Dalvi SS, Chogle AR, et al. Efficacy of a 14-day primaquine regimen in preventing relapses in patients with *Plasmodium vivax* malaria in Mumbai, India. Trans R Soc Trop Med Hyg. 2003;97(4):438-40. Epub 2004/07/21. PubMed PMID: 15259476.

89. Ruebush TK, 2nd, Zegarra J, Cairo J, Andersen EM, Green M, Pillai DR, et al. Chloroquine-resistant *Plasmodium vivax* malaria in Peru. Am J Trop Med Hyg. 2003;69(5):548-52. Epub 2003/12/26. PubMed PMID: 14695094.

90. Silachamroon U, Krudsood S, Treeprasertsuk S, Wilairatana P, Chalearmrult K, Mint HY, et al. Clinical trial of oral artesunate with or without high-dose primaquine for the treatment of vivax malaria in Thailand. Am J Trop Med Hyg. 2003;69(1):14-8. Epub 2003/08/23. PubMed PMID: 12932090; PubMed Central PMCID: PMCPMC3123523.

91. Valibayov A, Abdullayev F, Mammadov S, Gasimov E, Sabatinelli G, Kondrachine AV, et al. Clinical efficacy of chloroquine followed by primaquine for *Plasmodium vivax* treatment in Azerbaijan. Acta Trop. 2003;88(1):99-102. Epub 2003/08/29. PubMed PMID: 12943984.

92. Hamedi Y, Safa O, Zare S, Tan-ariya P, Kojima S, Looareesuwan S. Therapeutic efficacy of artesunate in *Plasmodium vivax* malaria in Thailand. Southeast Asian J Trop Med Public Health. 2004;35(3):570-4. Epub 2005/02/04. PubMed PMID: 15689068.

93. Hapuarachchi HA, Dayanath MY, Abeysundara S, Bandara KB, Abeyewickreme W, de Silva NR. Chloroquine resistant falciparum malaria among security forces personnel in the Northern Province of Sri Lanka. Ceylon Med J. 2004;49(2):47-51. Epub 2004/09/01. PubMed PMID: 15334798.

94. Kurcer MA, Simsek Z, Zeyrek FY, Atay S, Celik H, Kat I, et al. Efficacy of chloroquine in the treatment of *Plasmodium vivax* malaria in Turkey. Ann Trop Med Parasitol. 2004;98(5):447-51. Epub 2004/07/20. doi: 10.1179/000349804225021343. PubMed PMID: 15257793.

95. Leslie T, Rab MA, Ahmadzai H, Durrani N, Fayaz M, Kolaczinski J, et al. Compliance with 14-day primaquine therapy for radical cure of vivax malaria--a randomized placebo-controlled trial comparing unsupervised with supervised treatment. Trans R Soc Trop Med Hyg. 2004;98(3):168-73. Epub 2004/03/18. PubMed PMID: 15024927.

96. Vijaykadga S, Rojanawatsirivej C, Congpoung K, Wilairatana P, Satimai W, Uaekowitchai C, et al. Assessment of therapeutic efficacy of chloroquine for vivax malaria in Thailand. Southeast Asian J Trop Med Public Health. 2004;35(3):566-9. Epub 2005/02/04. PubMed PMID: 15689067.

97. Walsh DS, Wilairatana P, Tang DB, Heppner DG, Jr., Brewer TG, Krudsood S, et al. Randomized trial of 3-dose regimens of tafenoquine (WR238605) versus low-dose primaquine for preventing *Plasmodium vivax* malaria relapse. Clin Infect Dis. 2004;39(8):1095-103. Epub 2004/10/16. doi: 10.1086/424508. PubMed PMID: 15486831.

98. Dunne MW, Singh N, Shukla M, Valecha N, Bhattacharyya PC, Patel K, et al. A double-blind, randomized study of azithromycin compared to chloroquine for the treatment of *Plasmodium vivax* malaria in India. Am J Trop Med Hyg. 2005;73(6):1108-11. Epub 2005/12/16. PubMed PMID: 16354821.

99. Genton B, Baea K, Lorry K, Ginny M, Wines B, Alpers MP. Parasitological and clinical efficacy of standard treatment regimens against *Plasmodium falciparum*, *P. vivax* and *P. malariae* in Papua New Guinea. P N G Med J. 2005;48(3-4):141-50. Epub 2007/01/11. PubMed PMID: 17212060.

100. Khan SJ, Munib S. Efficacy of Halofantrine Hydrochloride in vivax malaria. J Postgraduate Medical Institute. 2005;19(3):276-80.

101. Yeramian P, Meshnick SR, Krudsood S, Chalermrut K, Silachamroon U, Tangpukdee N, et al. Efficacy of DB289 in Thai patients with *Plasmodium vivax* or acute, uncomplicated *Plasmodium falciparum* infections. J Infect Dis. 2005;192(2):319-22. Epub 2005/06/18. doi: 10.1086/430928. PubMed PMID: 15962227.

102. Alvarez G, Pineros JG, Tobon A, Rios A, Maestre A, Blair S, et al. Efficacy of three chloroquine-primaquine regimens for treatment of *Plasmodium vivax* malaria in Colombia. Am J Trop Med Hyg. 2006;75(4):605-9. Epub 2006/10/14. PubMed PMID: 17038680.

103. Khan MZ, Isani Z, Ahmed TM, Zafar AB, Gilal N, Maqbool S, et al. Efficacy and safety of halofantrine in Pakistani children and adults with malaria caused by *P. falciparum* and *P. vivax*. Southeast Asian J Trop Med Public Health. 2006;37(4):613-8. Epub 2006/11/24. PubMed PMID: 17121283.

104. Krudsood S, Wilairatana P, Tangpukdee N, Chalermrut K, Srivilairit S, Thanachartwet V, et al. Safety and tolerability of elubaquine (bulaquine, CDRI 80/53) for treatment of *Plasmodium vivax* malaria in Thailand. Korean J Parasitol. 2006;44(3):221-8. Epub 2006/09/14. PubMed PMID: 16969059; PubMed Central PMCID: PMCPMC2532664.

105. Kurcer MA, Simsek Z, Kurcer Z. The decreasing efficacy of chloroquine in the treatment of *Plasmodium vivax* malaria, in Sanliurfa, south-eastern Turkey. Ann Trop Med Parasitol. 2006;100(2):109-13. Epub 2006/02/24. doi: 10.1179/136485906X86284. PubMed PMID: 16492358.

106. Maguire JD, Krisin, Marwoto H, Richie TL, Fryauff DJ, Baird JK. Mefloquine is highly efficacious against chloroquine-resistant *Plasmodium vivax* malaria and *Plasmodium falciparum* malaria in Papua, Indonesia. Clin Infect Dis. 2006;42(8):1067-72. Epub 2006/04/01. doi: 10.1086/501357. PubMed PMID: 16575721.

107. Tasanor O, Ruengweerayut R, Sirichaisinthop J, Congpuong K, Wernsdorfer WH, Na-Bangchang K. Clinical-parasitological response and *in-vitro* sensitivity of *Plasmodium vivax* to chloroquine and quinine on the western border of Thailand. Trans R Soc Trop Med Hyg. 2006;100(5):410-8. Epub 2006/02/25. doi: 10.1016/j.trstmh.2005.04.024. PubMed PMID: 16497347.

108. Valecha N, Joshi H, Eapen A, Ravinderan J, Kumar A, Prajapati SK, et al. Therapeutic efficacy of chloroquine in *Plasmodium vivax* from areas with different epidemiological patterns in India and their *Pvdhfr* gene mutation pattern. Trans R Soc Trop Med Hyg. 2006;100(9):831-7. Epub 2006/03/04. doi: 10.1016/j.trstmh.2005.11.012. PubMed PMID: 16513151.

109. Dao NV, Cuong BT, Ngoa ND, Thuy le TT, The ND, Duy DN, et al. Vivax malaria: preliminary observations following a shorter course of treatment with artesunate plus primaquine. Trans R Soc Trop Med Hyg. 2007;101(6):534-9. Epub 2007/03/21. doi: 10.1016/j.trstmh.2007.01.003. PubMed PMID: 17368694.

110. de Santana Filho FS, Arcanjo AR, Chehuan YM, Costa MR, Martinez-Espinosa FE, Vieira JL, et al. Chloroquine-resistant *Plasmodium vivax*, Brazilian Amazon. Emerg Infect Dis. 2007;13(7):1125-6. Epub 2008/01/25. doi: 10.3201/eid1307.061386. PubMed PMID: 18214203; PubMed Central PMCID: PMCPMC2878224.

111. Kolaczinski K, Durrani N, Rahim S, Rowland M. Sulfadoxine-pyrimethamine plus artesunate compared with chloroquine for the treatment of vivax malaria in areas co-endemic for *Plasmodium falciparum* and *P. vivax*: a randomised non-inferiority trial in eastern Afghanistan. Trans R Soc Trop Med Hyg. 2007;101(11):1081-7. Epub 2007/08/21. doi: 10.1016/j.trstmh.2007.06.015. PubMed PMID: 17707447.

112. Leslie T, Mayan MI, Hasan MA, Safi MH, Klinkenberg E, Whitty CJ, et al. Sulfadoxine-pyrimethamine, chlorproguanil-dapsone, or chloroquine for the treatment of *Plasmodium vivax* malaria in Afghanistan and Pakistan: a randomized controlled trial. JAMA. 2007;297(20):2201-9. doi: 10.1001/jama.297.20.2201. PubMed PMID: 17519409.

113. Marfurt J, Mueller I, Sie A, Maku P, Goroti M, Reeder JC, et al. Low efficacy of amodiaquine or chloroquine plus sulfadoxine-pyrimethamine against *Plasmodium falciparum* and *P. vivax* malaria in Papua New Guinea. Am J Trop Med Hyg. 2007;77(5):947-54. Epub 2007/11/07. PubMed PMID: 17984359.

114. Nateghpour M, Sayedzadeh SA, Edrissian GH, Raeisi A, Jahantigh A, Motevalli-Haghi A, et al. Evaluation of Sensitivity of *Plasmodium vivax* to Chloroquine. Iran J Public Health. 2007;36(3):60-3.

115. Osorio L, Perez Ldel P, Gonzalez IJ. [Assessment of the efficacy of antimalarial drugs in Tarapaca, in the Colombian Amazon basin]. Biomedica. 2007;27(1):133-40. Epub 2007/06/05. doi: /S0120-41572007000100012. PubMed PMID: 17546230.

116. Barnadas C, Ratsimbasoa A, Tichit M, Bouchier C, Jahevitra M, Picot S, et al. *Plasmodium vivax* resistance to chloroquine in Madagascar: clinical efficacy and polymorphisms in *pvmdr1* and *pvcrt-o* genes. Antimicrob Agents Chemother. 2008;52(12):4233-40. Epub 2008/09/24. doi: 10.1128/AAC.00578-08. PubMed PMID: 18809933; PubMed Central PMCID: PMCPMC2592859.

117. Barnadas C, Tichit M, Bouchier C, Ratsimbasoa A, Randrianasolo L, Raherinjafy R, et al. *Plasmodium vivax dhfr* and *dhps* mutations in isolates from Madagascar and therapeutic response to sulphadoxine-pyrimethamine. Malar J. 2008;7:35. Epub 2008/02/28. doi: 10.1186/1475-2875-7-35. PubMed PMID: 18302746; PubMed Central PMCID: PMCPMC2268703.

118. Carmona-Fonseca J, Uscategui RM, Correa AM. Vivax malaria in children: Clinical features and response to chloroquine. [Spanish]. Colombia Medica. 2008;39(4):364-77.

119. Guthmann JP, Pittet A, Lesage A, Imwong M, Lindegardh N, Min Lwin M, et al. *Plasmodium vivax* resistance to chloroquine in Dawei, southern Myanmar. Trop Med Int Health. 2008;13(1):91-8. Epub 2008/02/23. doi: 10.1111/j.1365-3156.2007.01978.x. PubMed PMID: 18291007.

120. Krudsood S, Tangpukdee N, Wilairatana P, Phophak N, Baird JK, Brittenham GM, et al. High-dose primaquine regimens against relapse of *Plasmodium vivax* malaria. Am J Trop Med Hyg. 2008;78(5):736-40. Epub 2008/05/07. PubMed PMID: 18458306; PubMed Central PMCID: PMCPMC3129604.

121. Leslie T, Mayan I, Mohammed N, Erasmus P, Kolaczinski J, Whitty CJ, et al. A randomised trial of an eight-week, once weekly primaquine regimen to prevent relapse of *Plasmodium vivax* in Northwest Frontier Province, Pakistan. PLoS One. 2008;3(8):e2861. Epub 2008/08/07. doi: 10.1371/journal.pone.0002861. PubMed PMID: 18682739; PubMed Central PMCID: PMCPMC2481394.

122. Perez MA, Cortes LJ, Guerra AP, Knudson A, Usta C, Nicholls RS. [Efficacy of the amodiaquine+sulfadoxine-pyrimethamine combination and of chloroquine for the treatment of malaria in Cordoba, Colombia, 2006]. Biomedica. 2008;28(1):148-59. Epub 2008/07/23. PubMed PMID: 18645670.

123. Srivastava HC, Yadav RS, Joshi H, Valecha N, Mallick PK, Prajapati SK, et al. Therapeutic responses of *Plasmodium vivax* and *P. falciparum* to chloroquine, in an area of western India where *P. vivax* predominates. Ann Trop Med Parasitol. 2008;102(6):471-80. Epub 2008/09/11. doi: 10.1179/136485908X311759. PubMed PMID: 18782486.

124. Teka H, Petros B, Yamuah L, Tesfaye G, Elhassan I, Muchohi S, et al. Chloroquine-resistant *Plasmodium vivax* malaria in Debre Zeit, Ethiopia. Malar J. 2008;7:220. Epub 2008/10/31. doi: 10.1186/1475-2875-7-220. PubMed PMID: 18959774; PubMed Central PMCID: PMCPMC2584068.

125. Carmona-Fonseca J, Maestre A. Prevention of *Plasmodium vivax* malaria recurrence: efficacy of the standard total dose of primaquine administered over 3 days. Acta Trop. 2009;112(2):188-92. Epub 2009/08/06. doi: 10.1016/j.actatropica.2009.07.024. PubMed PMID: 19653988.

126. Hasugian AR, Tjitra E, Ratcliff A, Siswantoro H, Kenangalem E, Wuwung RM, et al. *In vivo* and *in vitro* efficacy of amodiaquine monotherapy for treatment of infection by chloroquine-resistant *Plasmodium vivax*. Antimicrob Agents Chemother. 2009;53(3):1094-9. Epub 2008/12/24. doi: 10.1128/AAC.01511-08. PubMed PMID: 19104023; PubMed Central PMCID: PMCPMC2650584.

127. Ketema T, Bacha K, Birhanu T, Petros B. Chloroquine-resistant *Plasmodium vivax* malaria in Serbo town, Jimma zone, south-west Ethiopia. Malar J. 2009;8:177. Epub 2009/08/01. doi: 10.1186/1475-2875-8-177. PubMed PMID: 19642976; PubMed Central PMCID: PMCPMC3224920.

128. Lee SW, Lee M, Lee DD, Kim C, Kim YJ, Kim JY, et al. Biological resistance of hydroxychloroquine for *Plasmodium vivax* malaria in the Republic of Korea. Am J Trop Med Hyg. 2009;81(4):600-4. Epub 2009/10/10. doi: 10.4269/ajtmh.2009.09-0102. PubMed PMID: 19815873.

129. Liang GL, Sun XD, Wang J, Zhang ZX. [Sensitivity of *Plasmodium vivax* to chloroquine in Laza City, Myanmar]. Zhongguo Ji Sheng Chong Xue Yu Ji Sheng Chong Bing Za Zhi. 2009;27(2):175-6. Epub 2009/10/27. PubMed PMID: 19856512.

130. Nateghpour M, Edrissian G, Torabi A, Raesi A, Motevalli-Haghi A, Abed-Khojasteh H, et al. Monitoring of *Plasmodium vivax* and *Plasmodium falciparum* response to chloroquine in Bandar-Abbas district, Hormozgan province, Iran. [Arabic]. Tehran Uni Med J. 2009;67(3):178-83.

131. Orjuela-Sanchez P, da Silva NS, da Silva-Nunes M, Ferreira MU. Recurrent parasitemias and population dynamics of *Plasmodium vivax* polymorphisms in rural Amazonia. Am J Trop Med Hyg. 2009;81(6):961-8. doi: 10.4269/ajtmh.2009.09-0337. PubMed PMID: 19996423.

132. Rogers WO, Sem R, Tero T, Chim P, Lim P, Muth S, et al. Failure of artesunate-mefloquine combination therapy for uncomplicated *Plasmodium falciparum* malaria in southern Cambodia. Malar J. 2009;8:10. Epub 2009/01/14. doi: 10.1186/1475-2875-8-10. PubMed PMID: 19138388; PubMed Central PMCID: PMCPMC2628668.

133. Sutanto I, Suprijanto S, Nurhayati, Manoempil P, Baird JK. Resistance to chloroquine by *Plasmodium vivax* at Alor in the Lesser Sundas Archipelago in eastern Indonesia. Am J Trop Med Hyg. 2009;81(2):338-42. Epub 2009/07/29. PubMed PMID: 19635895.

134. Carmona-Fonseca J. Vivax malaria in children: Recurrences with standard total dose of primaquine administered in 3 vs. 7 days Iatreia. 2010;23(1):10-20.

135. Congpuong K, Bualombai P, Banmairuroi V, Na-Bangchang K. Compliance with a three-day course of artesunate-mefloquine combination and baseline anti-malarial treatment in an area of Thailand with highly multidrug resistant falciparum malaria. Malar J. 2010;9:43. Epub 2010/02/06. doi: 10.1186/1475-2875-9-43. PubMed PMID: 20132537; PubMed Central PMCID: PMCPMC2829592.

136. Daneshvar C, Davis TM, Cox-Singh J, Rafa'ee MZ, Zakaria SK, Divis PC, et al. Clinical and parasitological response to oral chloroquine and primaquine in uncomplicated human *Plasmodium knowlesi* infections. Malar J. 2010;9:238. Epub 2010/08/21. doi: 10.1186/1475-2875-9-238. PubMed PMID: 20723228; PubMed Central PMCID: PMCPMC2933701.

137. Dilmec F, Kurcer MA, Akkafa F, Simsek Z. Monitoring of failure of chloroquine treatment for *Plasmodium vivax* using polymerase chain reaction in Sanliurfa province, Turkey. Parasitol Res. 2010;106(4):783-8. Epub 2010/02/09. doi: 10.1007/s00436-009-1710-8. PubMed PMID: 20140453.

138. Kinzer MH, Chand K, Basri H, Lederman ER, Susanti AI, Elyazar I, et al. Active case detection, treatment of falciparum malaria with combined chloroquine and sulphadoxine/pyrimethamine and vivax malaria with chloroquine and molecular markers of anti-malarial resistance in the Republic of Vanuatu. Malar J. 2010;9:89. Epub 2010/04/08. doi: 10.1186/1475-2875-9-89. PubMed PMID: 20370920; PubMed Central PMCID: PMCPMC2853556.

139. Pukrittayakamee S, Imwong M, Chotivanich K, Singhasivanon P, Day NP, White NJ. A comparison of two short-course primaquine regimens for the treatment and radical cure of *Plasmodium vivax* malaria in Thailand. Am J Trop Med Hyg. 2010;82(4):542-7. Epub 2010/03/30. doi: 10.4269/ajtmh.2010.09-0428. PubMed PMID: 20348496; PubMed Central PMCID: PMCPMC2844579.

140. Sutanto I, Endawati D, Ling LH, Laihad F, Setiabudy R, Baird JK. Evaluation of chloroquine therapy for vivax and falciparum malaria in southern Sumatra, western Indonesia. Malar J. 2010;9:52. doi: 10.1186/1475-2875-9-52. PubMed PMID: 20152016; PubMed Central PMCID: PMCPMC2831905.

141. Takeuchi R, Lawpoolsri S, Imwong M, Kobayashi J, Kaewkungwal J, Pukrittayakamee S, et al. Directly-observed therapy (DOT) for the radical 14-day primaquine treatment of *Plasmodium vivax* malaria on the Thai-Myanmar border. Malar J. 2010;9:308. Epub 2010/11/03. doi: 10.1186/1475-2875-9-308. PubMed PMID: 21040545; PubMed Central PMCID: PMCPMC2988041.

142. Yeshiwondim AK, Tekle AH, Dengela DO, Yohannes AM, Teklehaimanot A. Therapeutic efficacy of chloroquine and chloroquine plus primaquine for the treatment of *Plasmodium vivax* in Ethiopia. Acta Trop. 2010;113(2):105-13. Epub 2009/10/20. doi: 10.1016/j.actatropica.2009.10.001. PubMed PMID: 19835832.

143. Asih PB, Syafruddin D, Leake J, Sorontou Y, Sadikin M, Sauerwein RW, et al. Phenotyping clinical resistance to chloroquine in *Plasmodium vivax* in northeastern Papua, Indonesia. Int J Parasitol Drugs Drug Resist. 2011;1(1):28-32. Epub 2011/12/01. doi: 10.1016/j.ijpddr.2011.08.001. PubMed PMID: 24533261; PubMed Central PMCID: PMCPMC3898133.

144. Congpuon K, Satimai W, Sujariyakul A, Intanakom S, Harnpitakpong W, Pranuth Y, et al. *In vivo* sensitivity monitoring of chloroquine for the treatment of uncomplicated vivax malaria in four bordered provinces of Thailand during 2009-2010. J Vector Borne Dis. 2011;48(4):190-6. Epub 2012/02/03. PubMed PMID: 22297279.

145. Ketema T, Getahun K, Bacha K. Therapeutic efficacy of chloroquine for treatment of *Plasmodium vivax* malaria cases in Halaba district, South Ethiopia. Parasit Vectors. 2011;4:46. Epub 2011/04/02. doi: 10.1186/1756-3305-4-46. PubMed PMID: 21453465; PubMed Central PMCID: PMCPMC3076248.

146. Maneeboonyang W, Lawpoolsri S, Puangsa-Art S, Yimsamran S, Thanyavanich N, Wuthisen P, et al. Directly observed therapy with primaquine to reduce the recurrence rate of *Plasmodium vivax* infection along the Thai-Myanmar border. Southeast Asian J Trop Med Public Health. 2011;42(1):9-18. PubMed PMID: 21323159.

147. Muhamad P, Ruengweerayut R, Chacharoenkul W, Rungsihirunrat K, Na-Bangchang K. Monitoring of clinical efficacy and *in vitro* sensitivity of *Plasmodium vivax* to chloroquine in area along Thai Myanmar border during 2009-2010. Malar J. 2011;10:44. Epub 2011/02/18. doi: 10.1186/1475-2875-10-44. PubMed PMID: 21324161; PubMed Central PMCID: PMCPMC3055225.

148. Poravuth Y, Socheat D, Rueangweerayut R, Uthaisin C, Pyae Phyo A, Valecha N, et al. Pyronaridine-artesunate versus chloroquine in patients with acute *Plasmodium vivax* malaria: a randomized, double-blind, non-inferiority trial. PLoS One. 2011;6(1):e14501. doi: 10.1371/journal.pone.0014501. PubMed PMID: 21267072; PubMed Central PMCID: PMCPMC3022577.

149. Van den Eede P, Soto-Calle VE, Delgado C, Gamboa D, Grande T, Rodriguez H, et al. *Plasmodium vivax* sub-patent infections after radical treatment are common in Peruvian patients: results of a 1-year prospective cohort study. PLoS One. 2011;6(1):e16257. Epub 2011/02/08. doi: 10.1371/journal.pone.0016257. PubMed PMID: 21297986; PubMed Central PMCID: PMCPMC3030575.

150. Anez A, Navarro-Costa D, Yucra O, Garnica C, Melgar V, Moscoso M, et al. [Therapeutic response of *Plasmodium vivax* to chloroquine in Bolivia]. Biomedica. 2012;32(4):527-35. Epub 2013/05/30. doi: 10.1590/S0120-41572012000400008. PubMed PMID: 23715228.

151. Benjamin J, Moore B, Lee ST, Senn M, Griffin S, Lautu D, et al. Artemisinin-naphthoquine combination therapy for uncomplicated pediatric malaria: a tolerability, safety, and preliminary efficacy study. Antimicrob Agents Chemother. 2012;56(5):2465-71. Epub 2012/02/15. doi: 10.1128/AAC.06248-11. PubMed PMID: 22330921; PubMed Central PMCID: PMCPMC3346652.

152. Betuela I, Rosanas-Urgell A, Kiniboro B, Stanisic DI, Samol L, de Lazzari E, et al. Relapses contribute significantly to the risk of *Plasmodium vivax* infection and disease in Papua New Guinean children 1-5 years of age. J Infect Dis. 2012;206(11):1771-80. Epub 2012/09/12. doi: 10.1093/infdis/jis580. PubMed PMID: 22966124.

153. Graf PC, Durand S, Alvarez Antonio C, Montalvan C, Galves Montoya M, Green MD, et al. Failure of Supervised Chloroquine and Primaquine Regimen for the Treatment of *Plasmodium vivax* in the Peruvian Amazon. Malar Res Treat. 2012;2012:936067. Epub 2012/06/16. doi: 10.1155/2012/936067. PubMed PMID: 22701198; PubMed Central PMCID: PMCPMC3371340.

154. Heidari A, Keshavarz H, Shojaee S, Raeisi A, Dittrich S. In vivo Susceptibility of *Plasmodium vivax* to Chloroquine in Southeastern Iran. Iran J Parasitol. 2012;7(2):8-14. Epub 2012/10/31. PubMed PMID: 23109940; PubMed Central PMCID: PMCPMC3469182.

155. Mishra N, Singh JP, Srivastava B, Arora U, Shah NK, Ghosh SK, et al. Monitoring antimalarial drug resistance in India via sentinel sites: outcomes and risk factors for treatment failure, 2009-2010. Bull World Health Organ. 2012;90(12):895-904. Epub 2013/01/04. doi: 10.2471/BLT.12.109124. PubMed PMID: 23284195; PubMed Central PMCID: PMCPMC3524963.

156. Pedro RS, Guaraldo L, Campos DP, Costa AP, Daniel-Ribeiro CT, Brasil P. *Plasmodium vivax* malaria relapses at a travel medicine centre in Rio de Janeiro, a non-endemic area in Brazil. Malar J. 2012;11:245. Epub 2012/07/31. doi: 10.1186/1475-2875-11-245. PubMed PMID: 22839416; PubMed Central PMCID: PMCPMC3416703.

157. Saravu K, Acharya V, Kumar K, Kumar R. *Plasmodium vivax* remains responsive to chloroquine with primaquine treatment regimen: a prospective cohort study from tertiary care teaching hospital in southern India. Trop Doct. 2012;42(3):163-4. Epub 2012/04/21. doi: 10.1258/td.2012.120038. PubMed PMID: 22516030.

158. Ganguly S, Saha P, Guha SK, Das S, Bera DK, Biswas A, et al. *In vivo* therapeutic efficacy of chloroquine alone or in combination with primaquine against vivax malaria in Kolkata, West Bengal, India, and polymorphism in *pvmdr1* and *pvcrt-o* genes. Antimicrob Agents Chemother. 2013;57(3):1246-51. Epub 2012/12/25. doi: 10.1128/AAC.02050-12. PubMed PMID: 23262997; PubMed Central PMCID: PMCPMC3591881.

159. Liu H, Yang HL, Xu JW, Wang JZ, Nie RH, Li CF. Artemisinin-naphthoquine combination versus chloroquine-primaquine to treat vivax malaria: an open-label randomized and non-inferiority trial in Yunnan Province, China. Malar J. 2013;12:409. Epub 2013/11/13. doi: 10.1186/1475-2875-12-409. PubMed PMID: 24215565; PubMed Central PMCID: PMCPMC3833846.

160. Llanos-Cuentas A, Lacerda MV, Rueangweerayut R, Krudsood S, Gupta SK, Kochar SK, et al. Tafenoquine plus chloroquine for the treatment and relapse prevention of *Plasmodium vivax* malaria (DETECTIVE): a multicentre, double-blind, randomised, phase 2b dose-selection study. Lancet. 2014;383(9922):1049-58. doi: 10.1016/S0140-6736(13)62568-4. PubMed PMID: 24360369.

161. Manandhar S, Bhusal CL, Ghimire U, Singh SP, Karmacharya DB, Dixit SM. A study on relapse/re-infection rate of *Plasmodium vivax* malaria and identification of the predominant genotypes of *P. vivax* in two endemic districts of Nepal. Malar J. 2013;12:324. Epub 2013/09/18. doi: 10.1186/1475-2875-12-324. PubMed PMID: 24041296; PubMed Central PMCID: PMCPMC3848640.

162. Miahipour A, Keshavarz H, Heidari A, Raeisi A, Rezaeian M, Rezaei S. Assessment of the efficacy of 8 weeks of primaquine for the prevention of relapse in vivax malaria patients using SSCP-PCR and sequencing in South and South-East Iran, 2008-2011. Trans R Soc Trop Med Hyg. 2013;107(7):420-6. Epub 2013/05/23. doi: 10.1093/trstmh/trt035. PubMed PMID: 23694816.

163. Rios A, Alvarez G, Blair S. [Ten years of chloroquine efficacy for uncomplicated *Plasmodium vivax* malaria treatment, Turbo, Antioquia, 2002 and 2011]. Biomedica. 2013;33(3):429-38. Epub 2014/03/22. PubMed PMID: 24652179.

164. Zhu G, Lu F, Cao J, Zhou H, Liu Y, Han ET, et al. Blood stage of *Plasmodium vivax* in central China is still susceptible to chloroquine plus primaquine combination therapy. Am J Trop Med Hyg. 2013;89(1):184-7. Epub 2013/05/15. doi: 10.4269/ajtmh.12-0683. PubMed PMID: 23669232; PubMed Central PMCID: PMCPMC3748480.

165. Amaratunga C, Sreng S, Mao S, Tullo GS, Anderson JM, Chuor CM, et al. Chloroquine remains effective for treating *Plasmodium vivax* malaria in Pursat province, Western Cambodia. Antimicrob Agents Chemother. 2014;58(10):6270-2. Epub 2014/07/23. doi: 10.1128/AAC.03026-14. PubMed PMID: 25049249; PubMed Central PMCID: PMCPMC4187907.

166. Delgado-Ratto C, Soto-Calle VE, Van den Eede P, Gamboa D, Rosas A, Abatih EN, et al. Population structure and spatio-temporal transmission dynamics of *Plasmodium vivax* after radical cure treatment in a rural village of the Peruvian Amazon. Malar J. 2014;13:8. Epub 2014/01/08. doi: 10.1186/1475-2875-13-8. PubMed PMID: 24393454; PubMed Central PMCID: PMCPMC3893378.

167. Liu H, Yang HL, Tang LH, Li XL, Huang F, Wang JZ, et al. Monitoring *Plasmodium vivax* chloroquine sensitivity along China-Myanmar border of Yunnan Province, China during 2008-2013. Malar J. 2014;13:364. Epub 2014/09/17. doi: 10.1186/1475-2875-13-364. PubMed PMID: 25224069; PubMed Central PMCID: PMCPMC4180583.

168. Marques MM, Costa MR, Santana Filho FS, Vieira JL, Nascimento MT, Brasil LW, et al. *Plasmodium vivax* chloroquine resistance and anemia in the western Brazilian Amazon. Antimicrob Agents Chemother. 2014;58(1):342-7. Epub 2013/10/30. doi: 10.1128/AAC.02279-12. PubMed PMID: 24165179; PubMed Central PMCID: PMCPMC3910749.

169. Rajgor DD, Gogtay NJ, Kadam VS, Kocharekar MM, Parulekar MS, Dalvi SS, et al. Antirelapse Efficacy of Various Primaquine Regimens for *Plasmodium vivax*. Malar Res Treat. 2014;2014:347018. Epub 2014/10/09. doi: 10.1155/2014/347018. PubMed PMID: 25295216; PubMed Central PMCID: PMCPMC4176909.

170. Shalini S, Chaudhuri S, Sutton PL, Mishra N, Srivastava N, David JK, et al. Chloroquine efficacy studies confirm drug susceptibility of *Plasmodium vivax* in Chennai, India. Malar J. 2014;13:129. Epub 2014/04/02. doi: 10.1186/1475-2875-13-129. PubMed PMID: 24685286; PubMed Central PMCID: PMCPMC4021252.

171. Ould Ahmedou Salem MS, Mohamed Lemine YO, Deida JM, Lemrabott MA, Ouldabdallahi M, Ba MD, et al. Efficacy of chloroquine for the treatment of *Plasmodium vivax* in the Saharan zone in Mauritania. Malar J. 2015;14:39. Epub 2015/01/30. doi: 10.1186/s12936-015-0563-0. PubMed PMID: 25626475; PubMed Central PMCID: PMCPMC4318167.

172. Anez A, Moscoso M, Laguna A, Garnica C, Melgar V, Cuba M, et al. Resistance of infection by *Plasmodium vivax* to chloroquine in Bolivia. Malar J. 2015;14:261. Epub 2015/07/02. doi: 10.1186/s12936-015-0774-4. PubMed PMID: 26126708; PubMed Central PMCID: PMCPMC4488061.

173. Assefa M, Eshetu T, Biruksew A. Therapeutic efficacy of chloroquine for the treatment of *Plasmodium vivax* malaria among outpatients at Hossana Health Care Centre, southern Ethiopia. Malar J. 2015;14:458. Epub 2015/11/19. doi: 10.1186/s12936-015-0983-x. PubMed PMID: 26577669; PubMed Central PMCID: PMCPMC4650862.

174. Cheoymang A, Ruenweerayut R, Muhamad P, Rungsihirunrat K, Na-Bangchang K. Patients' adherence and clinical effectiveness of a 14-day course of primaquine when given with a 3-day chloroquine in patients with *Plasmodium vivax*

at the Thai-Myanmar border. Acta Trop. 2015;152:151-6. Epub 2015/08/19. doi: 10.1016/j.actatropica.2015.08.008. PubMed PMID: 26278026.

175. Getachew S, Thriemer K, Auburn S, Abera A, Gadisa E, Aseffa A, et al. Chloroquine efficacy for *Plasmodium vivax* malaria treatment in southern Ethiopia. Malar J. 2015;14:525. Epub 2015/12/26. doi: 10.1186/s12936-015-1041-4. PubMed PMID: 26702611; PubMed Central PMCID: PMCPMC4690314.

176. Gomes Mdo S, Vieira JL, Machado RL, Nacher M, Stefani A, Musset L, et al. Efficacy in the treatment of malaria by *Plasmodium vivax* in Oiapoque, Brazil, on the border with French Guiana: the importance of control over external factors. Malar J. 2015;14:402. Epub 2015/10/11. doi: 10.1186/s12936-015-0925-7. PubMed PMID: 26453152; PubMed Central PMCID: PMCPMC4600333.

177. Gonzalez-Ceron L, Rodriguez MH, Sandoval MA, Santillan F, Galindo-Virgen S, Betanzos AF, et al. Effectiveness of combined chloroquine and primaquine treatment in 14 days versus intermittent single dose regimen, in an open, non-randomized, clinical trial, to eliminate *Plasmodium vivax* in southern Mexico. Malar J. 2015;14:426. Epub 2015/11/01. doi: 10.1186/s12936-015-0938-2. PubMed PMID: 26518132; PubMed Central PMCID: PMCPMC4628368.

178. Pareek A, Chandurkar N, Gogtay N, Deshpande A, Kakrani A, Kaneria M, et al. Sustained Release Formulation of Primaquine for Prevention of Relapse of *Plasmodium vivax* Malaria: A Randomized, Double-Blind, Comparative, Multicentric Study. Malar Res Treat. 2015;2015:579864. Epub 2015/09/15. doi: 10.1155/2015/579864. PubMed PMID: 26366319; PubMed Central PMCID: PMCPMC4558454.

179. Rishikesh K, Kamath A, Hande MH, Vidyasagar S, Acharya RV, Acharya V, et al. Therapeutic assessment of chloroquine-primaquine combined regimen in adult cohort of *Plasmodium vivax* malaria from a tertiary care hospital in southwestern India. Malar J. 2015;14:310. Epub 2015/08/12. doi: 10.1186/s12936-015-0824-y. PubMed PMID: 26259839; PubMed Central PMCID: PMCPMC4532140.

180. Thanh PV, Hong NV, Van NV, Louisa M, Baird K, Xa NX, et al. Confirmed *Plasmodium vivax* Resistance to Chloroquine in Central Vietnam. Antimicrob Agents Chemother. 2015;59(12):7411-9. doi: 10.1128/AAC.00791-15. PubMed PMID: 26392501; PubMed Central PMCID: PMCPMC4649222.

181. Yuan L, Wang Y, Parker DM, Gupta B, Yang Z, Liu H, et al. Therapeutic responses of *Plasmodium vivax* malaria to chloroquine and primaquine treatment in northeastern Myanmar. Antimicrob Agents Chemother. 2015;59(2):1230-5. doi: 10.1128/AAC.04270-14. PubMed PMID: 25512415; PubMed Central PMCID: PMCPMC4335844.

182. Beyene HB, Beyene MB, Ebstie YA, Desalegn Z. Efficacy of Chloroquine for the Treatment of Vivax malaria in Northwest Ethiopia. PLoS One. 2016;11(8):e0161483. Epub 2016/09/01. doi: 10.1371/journal.pone.0161483. PubMed PMID: 27579480; PubMed Central PMCID: PMCPMC5007045.

183. Grigg MJ, William T, Menon J, Barber BE, Wilkes CS, Rajahram GS, et al. Efficacy of Artesunate-mefloquine for Chloroquine-resistant Plasmodium vivax Malaria in Malaysia: An Open-label, Randomized, Controlled Trial. Clin Infect Dis. 2016;62(11):1403-11. doi: 10.1093/cid/ciw121. PubMed PMID: 27107287; PubMed Central PMCID: PMCPMC4872287.

184. Lo E, Nguyen J, Oo W, Hemming-Schroeder E, Zhou G, Yang Z, et al. Examining *Plasmodium falciparum* and *P. vivax* clearance subsequent to antimalarial drug treatment in the Myanmar-China border area based on quantitative real-time polymerase chain reaction. BMC Infect Dis. 2016;16:154. Epub 2016/04/17. doi: 10.1186/s12879-016-1482-6. PubMed PMID: 27084511; PubMed Central PMCID: PMCPMC4833920.

185. Longley RJ, Sripoorote P, Chobson P, Saeseu T, Sukasem C, Phuanukoonnon S, et al. High Efficacy of Primaquine Treatment for *Plasmodium vivax* in Western Thailand. Am J Trop Med Hyg. 2016;95(5):1086-9. Epub 2016/11/04. doi: 10.4269/ajtmh.16-0410. PubMed PMID: 27601524; PubMed Central PMCID: PMCPMC5094221.

186. Mishra N, Srivastava B, Bharti RS, Rana R, Kaitholia K, Anvikar AR, et al. Monitoring the efficacy of antimalarial medicines in India via sentinel sites: Outcomes and risk factors for treatment failure. J Vector Borne Dis. 2016;53(2):168-78. Epub 2016/06/30. PubMed PMID: 27353588.

187. Moore BR, Benjamin JM, Auyeung SO, Salman S, Yadi G, Griffin S, et al. Safety, tolerability and pharmacokinetic properties of coadministered azithromycin and piperaquine in pregnant Papua New Guinean women. Br J Clin Pharmacol. 2016;82(1):199-212. Epub 2016/02/19. doi: 10.1111/bcp.12910. PubMed PMID: 26889763; PubMed Central PMCID: PMCPMC4917786.

188. Negreiros S, Farias S, Viana GM, Okoth SA, Chenet SM, de Souza TM, et al. Efficacy of Chloroquine and Primaquine for the Treatment of Uncomplicated *Plasmodium vivax* Malaria in Cruzeiro do Sul, Brazil. Am J Trop Med Hyg. 2016;95(5):1061-8. Epub 2016/11/04. doi: 10.4269/ajtmh.16-0075. PubMed PMID: 27549633; PubMed Central PMCID: PMCPMC5094218.

189. Pereira D, Daher A, Zanini G, Maia I, Fonseca L, Pitta L, et al. Safety, efficacy and pharmacokinetic evaluations of a new coated chloroquine tablet in a single-arm open-label non-comparative trial in Brazil: a step towards a user-friendly malaria vivax treatment. Malar J. 2016;15:477. Epub 2016/09/19. doi: 10.1186/s12936-016-1530-0. PubMed PMID: 27639847; PubMed Central PMCID: PMCPMC5027105.

190. Phyo AP, Jittamala P, Nosten FH, Pukrittayakamee S, Imwong M, White NJ, et al. Antimalarial activity of artefenomel (OZ439), a novel synthetic antimalarial endoperoxide, in patients with *Plasmodium falciparum* and *Plasmodium vivax* malaria: an open-label phase 2 trial. Lancet Infect Dis. 2016;16(1):61-9. Epub 2015/10/09. doi: 10.1016/S1473-3099(15)00320-5. PubMed PMID: 26448141; PubMed Central PMCID: PMCPMC4700386.

191. Saravu K, Kumar R, Ashok H, Kundapura P, Kamath V, Kamath A, et al. Therapeutic Assessment of Chloroquine-Primaquine Combined Regimen in Adult Cohort of *Plasmodium vivax* Malaria from Primary Care Centres in Southwestern India. PLoS One. 2016;11(6):e0157666. Epub 2016/06/18. doi: 10.1371/journal.pone.0157666. PubMed PMID: 27315280; PubMed Central PMCID: PMCPMC4912090.

192. Valecha N, Savargaonkar D, Srivastava B, Rao BH, Tripathi SK, Gogtay N, et al. Comparison of the safety and efficacy of fixed-dose combination of arterolane maleate and piperaquine phosphate with chloroquine in acute, uncomplicated *Plasmodium vivax* malaria: a phase III, multicentric, open-label study. Malar J. 2016;15:42. Epub 2016/01/29. doi: 10.1186/s12936-016-1084-1. PubMed PMID: 26818020; PubMed Central PMCID: PMCPMC4728808.

193. Wangchuk S, Drukpa T, Penjor K, Peldon T, Dorjey Y, Dorji K, et al. Where chloroquine still works: the genetic make-up and susceptibility of *Plasmodium vivax* to chloroquine plus primaquine in Bhutan. Malar J. 2016;15(1):277. Epub 2016/05/14. doi: 10.1186/s12936-016-1320-8. PubMed PMID: 27176722; PubMed Central PMCID: PMCPMC4866075.

194. Waqar T, Khushdil A, Haque K. Efficacy of Chloroquine as a first line agent in the treatment of uncomplicated malaria due to *Plasmodium vivax* in children and treatment practices in Pakistan: A Pilot study. J Pak Med Assoc. 2016;66(1):30-3. Epub 2015/12/30. PubMed PMID: 26712176.

195. White NJ, Duong TT, Uthaisin C, Nosten F, Phyo AP, Hanboonkunupakarn B, et al. Antimalarial Activity of KAF156 in Falciparum and Vivax Malaria. N Engl J Med. 2016;375(12):1152-60. Epub 2016/09/23. doi: 10.1056/NEJMoa1602250. PubMed PMID: 27653565; PubMed Central PMCID: PMCPMC5142602.

196. Zuluaga-Idarraga L, Blair S, Akinyi Okoth S, Udhayakumar V, Marcet PL, Escalante AA, et al. Prospective Study of *Plasmodium vivax* Malaria Recurrence after Radical Treatment with a Chloroquine-Primaquine Standard Regimen in Turbo, Colombia. Antimicrob Agents Chemother. 2016;60(8):4610-9. Epub 2016/05/18. doi: 10.1128/AAC.00186-16. PubMed PMID: 27185794; PubMed Central PMCID: PMCPMC4958195.

197. Awab GR, Imwong M, Bancone G, Jeeyapant A, Day NPJ, White NJ, et al. Chloroquine-Primaquine versus Chloroquine Alone to Treat Vivax Malaria in Afghanistan: An Open Randomized Superiority Trial. Am J Trop Med Hyg. 2017;97(6):1782-7. Epub 2017/11/17. doi: 10.4269/ajtmh.17-0290. PubMed PMID: 29141719; PubMed Central PMCID: PMCPMC5805052.

198. Dharmawardena P, Rodrigo C, Mendis K, de AWGW, Premaratne R, Ringwald P, et al. Response of imported malaria patients to antimalarial medicines in Sri Lanka following malaria elimination. PLoS One. 2017;12(11):e0188613. Epub 2017/11/29. doi: 10.1371/journal.pone.0188613. PubMed PMID: 29182619; PubMed Central PMCID: PMCPMC5705151.

199. Fukuda MM, Krudsood S, Mohamed K, Green JA, Warrasak S, Noedl H, et al. A randomized, double-blind, active-control trial to evaluate the efficacy and safety of a three day course of tafenoquine monotherapy for the treatment of *Plasmodium vivax* malaria. PLoS One. 2017;12(11):e0187376. Epub 2017/11/10. doi: 10.1371/journal.pone.0187376. PubMed PMID: 29121061; PubMed Central PMCID: PMCPMC5679603.

200. Htun MW, Mon NCN, Aye KM, Hlaing CM, Kyaw MP, Handayuni I, et al. Chloroquine efficacy for *Plasmodium vivax* in Myanmar in populations with high genetic diversity and moderate parasite gene flow. Malar J. 2017;16(1):281. Epub 2017/07/12. doi: 10.1186/s12936-017-1912-y. PubMed PMID: 28693552; PubMed Central PMCID: PMCPMC5504659.

201. Mac Donald-Ottevanger MS, Adhin MR, Jitan JK, Bretas G, Vreden SG. Primaquine double dose for 7 days is inferior to single-dose treatment for 14 days in preventing *Plasmodium vivax* recurrent episodes in Suriname. Infect Drug Resist. 2018;11:3-8. Epub 2018/01/11. doi: 10.2147/IDR.S135897. PubMed PMID: 29317838; PubMed Central PMCID: PMCPMC5743107.

202. Nyunt MH, Han JH, Wang B, Aye KM, Aye KH, Lee SK, et al. Clinical and molecular surveillance of drug resistant vivax malaria in Myanmar (2009-2016). Malar J. 2017;16(1):117. Epub 2017/03/17. doi: 10.1186/s12936-017-1770-7. PubMed PMID: 28298235; PubMed Central PMCID: PMCPMC5353783.

203. Seifu S, Zeynudin A, Zemene E, Suleman S, Biruksew A. Therapeutic efficacy of chloroquine for the treatment of *Plasmodium vivax* malaria among outpatients at Shawa Robit Health Care Centre, North-East Ethiopia. Acta Trop. 2017;171:44-51. Epub 2017/03/17. doi: 10.1016/j.actatropica.2017.02.027. PubMed PMID: 28300558.

204. Shaikh S, Memon S, Das C. Efficacy of oral chloroquine in uncomplicated vivax malaria in children. Medical Forum Monthly. 2017;28(4):148-51.

205. Siqueira AM, Alencar AC, Melo GC, Magalhaes BL, Machado K, Alencar Filho AC, et al. Fixed-Dose Artesunate-Amodiaquine Combination vs Chloroquine for Treatment of Uncomplicated Blood Stage *P. vivax* Infection in the Brazilian Amazon: An Open-Label Randomized, Controlled Trial. Clin Infect Dis. 2017;64(2):166-74. Epub 2016/12/19. doi: 10.1093/cid/ciw706. PubMed PMID: 27988484; PubMed Central PMCID: PMCPMC5215218.

206. Macareo L, Lwin KM, Cheah PY, Yuentrakul P, Miller RS, Nosten F. Triangular test design to evaluate tinidazole in the prevention of *Plasmodium vivax* relapse. Malar J. 2013;12:173. Epub 2013/05/31. doi: 10.1186/1475-2875-12-173. PubMed PMID: 23718705; PubMed Central PMCID: PMCPMC3671156.

207. Kheng S, Muth S, Taylor WR, Tops N, Kosal K, Sothea K, et al. Tolerability and safety of weekly primaquine against relapse of *Plasmodium vivax* in Cambodians with glucose-6-phosphate dehydrogenase deficiency. BMC Med. 2015;13:203. Epub 2015/08/26. doi: 10.1186/s12916-015-0441-1. PubMed PMID: 26303162; PubMed Central PMCID: PMCPMC4549079.

208. Eibach D, Ceron N, Krishnalall K, Carter K, Bonnot G, Bienvenu AL, et al. Therapeutic efficacy of artemether-lumefantrine for *Plasmodium vivax* infections in a prospective study in Guyana. Malar J. 2012;11:347. Epub 2012/10/23. doi: 10.1186/1475-2875-11-347. PubMed PMID: 23083017; PubMed Central PMCID: PMCPMC3488321.

209. Senn N, Rarau P, Manong D, Salib M, Siba P, Reeder JC, et al. Effectiveness of artemether/lumefantrine for the treatment of uncomplicated *Plasmodium vivax* and *P. falciparum* malaria in young children in Papua New Guinea. Clin Infect Dis. 2013;56(10):1413-20. Epub 2013/02/14. doi: 10.1093/cid/cit068. PubMed PMID: 23403171.

210. Leang R, Barrette A, Bouth DM, Menard D, Abdur R, Duong S, et al. Efficacy of dihydroartemisinin-piperaquine for treatment of uncomplicated *Plasmodium falciparum* and *Plasmodium vivax* in Cambodia, 2008 to 2010. Antimicrob Agents Chemother. 2013;57(2):818-26. Epub 2012/12/05. doi: 10.1128/AAC.00686-12. PubMed PMID: 23208711; PubMed Central PMCID: PMCPMC3553743.

211. Lon C, Manning JE, Vanachayangkul P, So M, Sea D, Se Y, et al. Efficacy of two versus three-day regimens of dihydroartemisinin-piperaquine for uncomplicated malaria in military personnel in northern Cambodia: an open-label randomized trial. PLoS One. 2014;9(3):e93138. Epub 2014/03/29. doi: 10.1371/journal.pone.0093138. PubMed PMID: 24667662; PubMed Central PMCID: PMCPMC3965521.

212. Krudsood S, Tangpukdee N, Muangnoicharoen S, Thanachartwet V, Luplertlop N, Srivilairit S, et al. Clinical efficacy of chloroquine versus artemether-lumefantrine for *Plasmodium vivax* treatment in Thailand. Korean J Parasitol. 2007;45(2):111-4. Epub 2007/06/16. PubMed PMID: 17570973; PubMed Central PMCID: PMCPMC2526312.

213. Yohannes AM, Teklehaimanot A, Bergqvist Y, Ringwald P. Confirmed vivax resistance to chloroquine and effectiveness of artemether-lumefantrine for the treatment of vivax malaria in Ethiopia. Am J Trop Med Hyg. 2011;84(1):137-40. Epub 2011/01/08. doi: 10.4269/ajtmh.2011.09-0723. PubMed PMID: 21212216; PubMed Central PMCID: PMCPMC3005510.

214. Tjitra E, Hasugian AR, Siswantoro H, Prasetyorini B, Ekowatiningsih R, Yusnita EA, et al. Efficacy and safety of artemisinin-naphthoquine versus dihydroartemisinin-piperaquine in adult patients with uncomplicated malaria: a multi-centre study in Indonesia. Malar J. 2012;11:153. Epub 2012/05/05. doi: 10.1186/1475-2875-11-153. PubMed PMID: 22554203; PubMed Central PMCID: PMCPMC3464686.

215. Shaikh S, Ahmed I, Memon SM, Saleem A, Memon H, Babar A. Therapeutic efficacy and safety of Dihydroartemisinin-piperaquine (DP) for the treatment of uncomplicated *Plasmodium vivax* malaria: A single center study. J Liaquat Uni Med Health Sci. 2017;16(2):93-8.
