# Supplementary material for: The efficacy of dihydroartemisinin-piperaquine and artemether-lumefantrine with and without primaquine on Plasmodium vivax recurrence: A systematic review and individual patient data meta-analysis
Source: PLoS Med. 2019 Oct 4;16(10):e1002928. doi: 10.1371/journal.pmed.1002928 (PMC6777759; doi:10.1371/journal.pmed.1002928)
Supplement: S1 Fig — (PDF) [file pmed.1002928.s004.pdf]

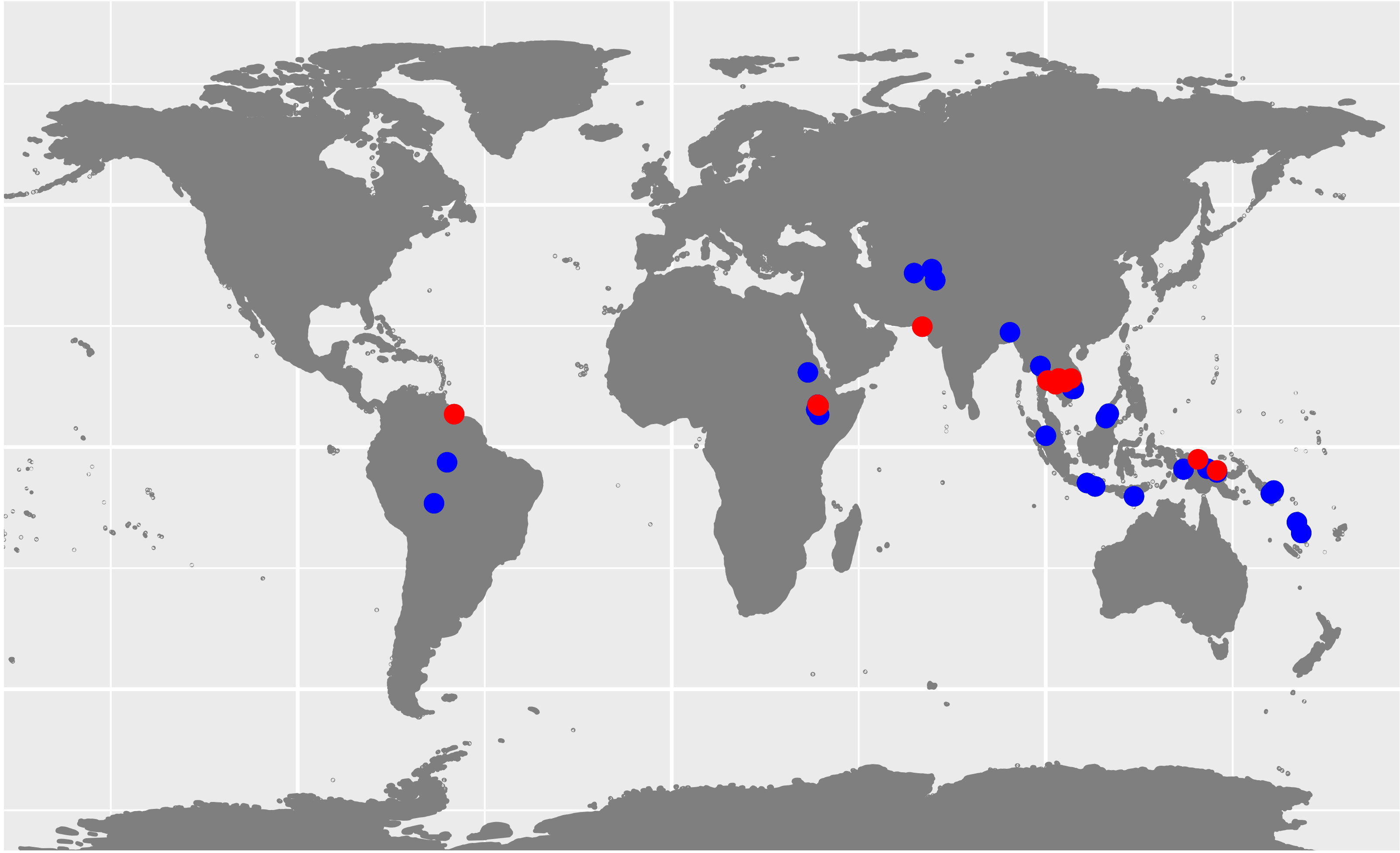

S1 Fig. Study sites for clinical trials

Blue – included studies; Red – targeted but not included; created in R using ggplot2 (H. Wickham. ggplot2: elegant graphics for data analysis. Springer New York, 2009.)
